# Household and personal air pollution exposure measurements from 120 communities in eight countries: results from the PURE-AIR study

**DOI:** 10.1016/S2542-5196(20)30197-2

**Published:** 2020-10

**Authors:** Matthew Shupler, Perry Hystad, Aaron Birch, Daniel Miller-Lionberg, Matthew Jeronimo, Raphael E Arku, Yen Li Chu, Maha Mushtaha, Laura Heenan, Sumathy Rangarajan, Pamela Seron, Fernando Lanas, Fairuz Cazor, Patricio Lopez-Jaramillo, Paul A Camacho, Maritza Perez, Karen Yeates, Nicola West, Tatenda Ncube, Brian Ncube, Jephat Chifamba, Rita Yusuf, Afreen Khan, Bo Hu, Xiaoyun Liu, Li Wei, Lap Ah Tse, Deepa Mohan, Parthiban Kumar, Rajeev Gupta, Indu Mohan, K G Jayachitra, Prem K Mony, Kamala Rammohan, Sanjeev Nair, P V M Lakshmi, Vivek Sagar, Rehman Khawaja, Romaina Iqbal, Khawar Kazmi, Salim Yusuf, Michael Brauer

**Affiliations:** School of Population and Public Health, University of British Columbia, Vancouver, BC, Canada; Department of Public Health and Policy, University of Liverpool, Liverpool, UK; College of Public Health and Human Sciences, Oregon State University, Corvallis, OR, USA; School of Population and Public Health, University of British Columbia, Vancouver, BC, Canada; Access Sensors Technologies, Fort Collins, CO, USA; School of Population and Public Health, University of British Columbia, Vancouver, BC, Canada; School of Population and Public Health, University of British Columbia, Vancouver, BC, Canada; School of Public Health and Health Sciences, University of Massachusetts Amherst, Amherst, MA, USA; School of Population and Public Health, University of British Columbia, Vancouver, BC, Canada; Population Health Research Institute, Hamilton Health Sciences, McMaster University, Hamilton, ON, Canada; Population Health Research Institute, Hamilton Health Sciences, McMaster University, Hamilton, ON, Canada; Population Health Research Institute, Hamilton Health Sciences, McMaster University, Hamilton, ON, Canada; Universidad de La Frontera, Temuco, Chile; Universidad de La Frontera, Temuco, Chile; Universidad de La Frontera, Temuco, Chile; Universidad de Santander (UDES), Bucaramanga, Colombia; FOSCAL, Floridablanca, Colombia; Universidad Militar Nueva Granada, Bogota, Colombia; Pamoja Tunaweza Research Centre, Moshi, Tanzania; Department of Medicine, Queen’s University, Kingston, ON, Canada; Pamoja Tunaweza Research Centre, Moshi, Tanzania; Department of Physiology, University of Zimbabwe, Harare, Zimbabwe; Department of Physiology, University of Zimbabwe, Harare, Zimbabwe; Department of Physiology, University of Zimbabwe, Harare, Zimbabwe; School of Life Sciences, Independent University, Dhaka, Bangladesh; School of Life Sciences, Independent University, Dhaka, Bangladesh; Medical Research & Biometrics Center, National Center for Cardiovascular Diseases, Fuwai Hospital, Chinese Academy of Medical Sciences, Beijing, China; Medical Research & Biometrics Center, National Center for Cardiovascular Diseases, Fuwai Hospital, Chinese Academy of Medical Sciences, Beijing, China; Medical Research & Biometrics Center, National Center for Cardiovascular Diseases, Fuwai Hospital, Chinese Academy of Medical Sciences, Beijing, China; Jockey Club School of Public health and Primary Care, the Chinese University of Hong Kong, Hong Kong Special Administrative Region, China; Madras Diabetes Research Foundation, Chennai, India; Madras Diabetes Research Foundation, Chennai, India; Eternal Heart Care Centre & Research Institute, Jaipur, India; Mahatma Gandhi Medical College, Jaipur, India; St John’s Medical College & Research Institute, Bangalore, India; St John’s Medical College & Research Institute, Bangalore, India; Health Action By People, Thiruvananthapuram and Medical College, Trivandrum, India; Health Action By People, Thiruvananthapuram and Medical College, Trivandrum, India; Post Graduate Institute of Medical Education and Research, Chandigarh, India; Post Graduate Institute of Medical Education and Research, Chandigarh, India; Department of Community Health Science, Aga Khan University Hospital, Karachi, Pakistan; Department of Community Health Science, Aga Khan University Hospital, Karachi, Pakistan; Department of Community Health Science, Aga Khan University Hospital, Karachi, Pakistan; Population Health Research Institute, Hamilton Health Sciences, McMaster University, Hamilton, ON, Canada; School of Population and Public Health, University of British Columbia, Vancouver, BC, Canada

## Abstract

**Background:**

Approximately 2·8 billion people are exposed to household air pollution from cooking with polluting fuels. Few monitoring studies have systematically measured health-damaging air pollutant (ie, fine particulate matter [PM_2·5_] and black carbon) concentrations from a wide range of cooking fuels across diverse populations. This multinational study aimed to assess the magnitude of kitchen concentrations and personal exposures to PM_2·5_ and black carbon in rural communities with a wide range of cooking environments.

**Methods:**

As part of the Prospective Urban and Rural Epidemiological (PURE) cohort, the PURE-AIR study was done in 120 rural communities in eight countries (Bangladesh, Chile, China, Colombia, India, Pakistan, Tanzania, and Zimbabwe). Data were collected from 2541 households and from 998 individuals (442 men and 556 women). Gravimetric (or filter-based) 48 h kitchen and personal PM_2·5_ measurements were collected. Light absorbance (10^−5^m^−1^) of the PM_2·5_ filters, a proxy for black carbon concentrations, was calculated via an image-based reflectance method. Surveys of household characteristics and cooking patterns were collected before and after the 48 h monitoring period.

**Findings:**

Monitoring of household air pollution for the PURE-AIR study was done from June, 2017, to September, 2019. A mean PM_2·5_ kitchen concentration gradient emerged across primary cooking fuels: gas (45 μg/m^3^ [95% CI 43–48]), electricity (53 μg/m^3^ [47–60]), coal (68 μg/m^3^ [61–77]), charcoal (92 μg/m^3^ [58–146]), agricultural or crop waste (106 μg/m^3^ [91–125]), wood (109 μg/m^3^ [102–118]), animal dung (224 μg/m^3^ [197–254]), and shrubs or grass (276 μg/m^3^ [223–342]). Among households cooking primarily with wood, average PM_2·5_ concentrations varied ten-fold (range: 40–380 μg/m^3^). Fuel stacking was prevalent (981 [39%] of 2541 households); using wood as a primary cooking fuel with clean secondary cooking fuels (eg, gas) was associated with 50% lower PM_2·5_ and black carbon concentrations than using only wood as a primary cooking fuel. Similar average PM_2·5_ personal exposures between women (67 μg/m^3^ [95% CI 62–72]) and men (62 [58–67]) were observed. Nearly equivalent average personal exposure to kitchen exposure ratios were observed for PM_2·5_ (0·79 [95% 0·71–0·88] for men and 0·82 [0·74–0·91] for women) and black carbon (0·64 [0·45–0·92] for men and 0·68 [0·46–1·02] for women).

**Interpretation:**

Using clean primary fuels substantially lowers kitchen PM_2·5_ concentrations. Importantly, average kitchen and personal PM_2·5_ measurements for all primary fuel types exceeded WHO’s Interim Target-1 (35 μg/m^3^ annual average), highlighting the need for comprehensive pollution mitigation strategies.

## Introduction

Approximately 2·8 billion people used polluting fuels (eg, solid fuels such as wood and coal, and kerosene) for cooking or heating, or both, in 2018 and were exposed to health-damaging levels of household air pollution.^1^ Exposure to elevated concentrations of fine particulate matter (PM_2·5_) is associated with a range of adverse health effects.^2-6^ The Global Burden of Diseases, Injuries, and Risk Factors Study (GBD) 2018 estimated that 1·6 million deaths were attributable to PM_2·5_ exposure from household air pollution in 2017.^7^ Additionally, household air pollution contributes to outdoor air pollution^8^ and black carbon, the second largest contributor to global warming.^9^

Few large-scale, systematic household air pollution measurement studies have included household concentrations and personal exposures of PM_2·5_ and black carbon. A pooled model of 2208 measurements from 44 studies in 13 countries from 1996 to 2017^10^ showed low precision in 24 h mean household PM_2·5_ concentrations across primary fuel types: gas or electric (100 μg/m^3^ [95% CI 40–270]), coal (320 μg/m^3^ [120–840]), traditional wood (400 μg/m^3^ [150–1040]), and animal dung (960 μg/m^3^ [360–2500]).^11^ Studies included in the model were typically done in few households (2–470 households; median 17) with diverse measurement methods.^10^ For logistical and financial reasons, most household air pollution studies have only collected kitchen concentrations; studies that collected personal measurements have typically monitored female exposures (ie, the main household cook) only.^11^ As the magnitudes of PM_2·5_ and black carbon exposures remain imprecise, substantial uncertainties remain in our epidemiological understanding of household air pollution.^8^ Large-scale household air pollution measurements in previously unmonitored communities will enable refined characterisation of exposure levels, which can improve future assessments of the effectiveness of household air pollution interventions (eg, the Household Air Pollution Intervention Tool [HAPIT]^12^) in improving health outcomes, estimates of disease burden due to household air pollution, and polices to reduce household air pollution exposures.

A multinational household air pollution monitoring study was implemented in 120 rural communities in eight countries from the pre-existing Prospective Urban and Rural Epidemiological (PURE) study. Household air pollution monitoring included integrated 48 h measurements of PM_2·5_ and black carbon alongside survey data on household and cooking characteristics that might influence household air pollution exposures, to provide important information on household and personal PM_2·5_ and black carbon exposures, including variations across diverse populations, and a range of cooking environment factors (eg, primary and secondary fuels used, and stove type).

## Methods

### Study design

The PURE-AIR study is nested within the larger PURE cohort, which includes around 200 000 participants from 26 high-income, middle-income, and low-income countries.^13^ In each country, participants were recruited from rural and urban communities clustered around urban centres (referred to as subnational regions) with access to laboratory equipment for processing of biological samples (for a list of subnational regions see the appendix p 9). Rural communities represent villages more than 50 km away from urban centres or without easy access to commuter transportation at baseline, but within a 45 min drive of a laboratory.^13^ Door-to-door convenience sampling was done in all PURE communities. Within communities, recruited participants were representative of the age and sex distribution of adults aged 35–70 years. Evaluation studies have shown age, sex, education, and mortality distributions of PURE participants to generally represent national statistics.^14^

The PURE-AIR study was done in 120 rural communities in eight low-income and middle-income PURE countries (Bangladesh [16 communities], Chile [three], China [38], Colombia [18], India [32], Pakistan [six], Tanzania [five], and Zimbabwe [two]) where more than 10% of households used polluting fuels (wood, animal dung, agricultural waste, coal, charcoal, shrubs or grass, and kerosene) at baseline; these classifications were based on World Bank data during PURE study commencement (2003).^15^ As a high amount of primary cooking fuel switching occurred between baseline assessment (which varied between countries; appendix p 2) and PURE-AIR monitoring,^16^ communities were strategically selected for household air pollution monitoring to ensure a sufficient distribution of polluting fuel types among household samples. Although study recruitment included a higher proportion of households using clean primary fuels compared with baseline (appendix p 3), stratified sampling by community-level baseline primary cooking fuel use statistics (eg, 60% wood, 40% liquefied petroleum gas, hereafter referred to as gas) was maintained to ensure variations in polluting cooking fuel types.

### Monitoring methods

Monitoring occurred from June, 2017, to September, 2019, by use of a standard protocol, as described elsewhere.^15^ Briefly, PM_2·5_ filter samples were collected with the ultrasonic personal Aerosol Sampler (UPAS; Access Sensor Technologies, Fort Collins, CO, USA) operated at a flow rate of 1·0 L/min and 50% duty cycle. The UPAS device was placed on a stand, approximately 1 m high and 1 m from the primary cookstove for 48 h kitchen monitoring. The 48 h sampling period was selected to capture potential day-to-day variation in household air pollution concentrations, while minimising monitoring costs and participant burden. In two regions of India and China, two 48 h kitchen samples were collected simultaneously in 26 households to evaluate variability in UPAS measurements. Previous laboratory evaluations and pilot studies^15,17,18^ have shown high correlation (*r*≥0·9) between the UPAS and well established filter-based monitors. All filters (including 269 blank filters—approximately 10% of household samples) were weighed before and after the sampling period for PM_2·5_ mass (method detection limit: 8·7 μg/m^3^; analytical limit of detection 1·2 μg/m^3^) with the same fully automated robotic balance system (Measurement Technology Laboratories, Bloomington, MN, USA) maintained in a temperature-controlled and humidity-controlled laboratory in Vancouver, BC, Canada (see appendix p 15 for details). Field blank filters were stored in research offices within the respective communities for the sampling duration, then packaged with sampled filters and shipped back to Canada for analysis. The absorption coefficient (light absorbance; 10^−5^m^−1^) of the PM_2·5_ filters weighed after sampling (method detection limit 0·47 10^−5^m^−1^), used as a proxy for black carbon concentrations,^19^ was calculated via a low-cost and evaluated image-based reflectance method.^20^ The image-based reflectance method was highly correlated (*r*^2^=0·99) with elemental carbon concentrations on sampled filters (1 absorbance unit [1×10^−5^m^−1^] is equivalent to 1·67 μg/m^3^ elemental carbon).^20^

In a subset of households (696 [27%] of 2541), 48 h personal sampling was done (simultaneously with kitchen monitoring), with the UPAS worn in an armband (787 [79%] of 998 samples) or harness (211 [21%] of 998 samples) at participants’ discretion. GPS data collected from the UPAS were used to evaluate the proportion of time participants spent away (>25 m radius) from their households during personal monitoring. Convenience sampling was used to select participants for personal monitoring; men and women from households selected for kitchen monitoring were sampled until the target sample size was achieved for each sex in the community (priority was given to paired male–female measurements from the same households). Before monitoring, a PURE-AIR survey was completed that contained the same cooking environment questions as a baseline PURE household survey, with additional questions on secondary fuel and stove type. After the 48 h monitoring period, another survey was completed on cooking and heating practices specific to the sampling period.^15^ Log files of flow volume and run-time were transferred to a central project server and an R program code automatically scanned files every 24 h to detect potential errors (eg, flow rate <0·5 L/min, sample time <43 h). Erroneous files were brought to the attention of the field team for 48 h remonitoring of households or individuals, or both.

### Statistical analysis

This descriptive analysis was focused on characterising multinational variations in concentrations and exposures by primary and secondary cooking fuel type. Household heating was also examined in six PURE-AIR subnational regions where heating fuel type varied among households using the same primary cooking fuel type. Seasonality, dichotomised as summer (April to September) or winter (October to March), and reversed for the southern hemisphere (ie, Chile, Tanzania, and Zimbabwe), was examined in subnational regions where more than 85% of samples were done in a single season, and via repeat measurements done approximately 6 months apart in 24 households in China (Beijing and Liaoning) and India (Chennai and Jaipur).

Descriptive statistics of measurements by primary cooking fuels used during monitoring are presented by key household characteristics (kitchen type, heating fuel, and fuel stacking), individual behaviours (cooking time, smoking status, and occupational exposure), and country or subnational region. All black carbon and PM_2·5_ measurements were log-transformed when generating summary statistics; geometric means (hereafter referred to as means) and 95% CIs were reported (significance was assessed via non-overlapping confidence intervals). Linear regression was used to characterise the relationship between PM_2·5_ and black carbon measurements for potential utility in estimating black carbon absorbance based on PM_2·5_ concentrations; Spearman’s correlation coefficients (*r*) are reported. Male-to-female and personal-to-kitchen PM_2·5_ and black carbon ratios are presented for 227 households with paired male–female samples (n=454) to better compare sex-specific exposures. All analyses were done in R, version 3.4.4.

### Role of the funding source

The funders of the study had no role in study design, data collection, data analysis, data interpretation, or writing of the report. The corresponding author had full access to the study datasets and was responsible for the decision to submit for publication.

## Results

Valid 48 h kitchen measurements were collected in more than 80% of attempts, leading to a final sample of 2541 households. GPS data obtained from the UPAS revealed that 45 (5%) of 998 participants did not travel for more than a 25 m radius away from their household during 48 h sampling (appendix p 34), suggesting potentially high compliance. Re-sampling occurred in 115 (5%) of 2541 households. Common monitoring issues were a depleted battery due to insufficient charging (154 [50%] of 308 errors), SD card tampering (68 [22%] of 308 errors), highly loaded filters (34 [11%] of 308 errors), and operating in extremely hot environments (nine [3%] of 308 errors). Duplicate 48 h kitchen samples from 25 households in India (n=11), China (n=9), and Pakistan (n=5) showed high agreement (*r*=0·8; p<0·0001; appendix p 24), with a median PM_2·5_ concentration difference of 8·5 μg/m^3^ (percentage difference 12·5%).

**Figure 1:**
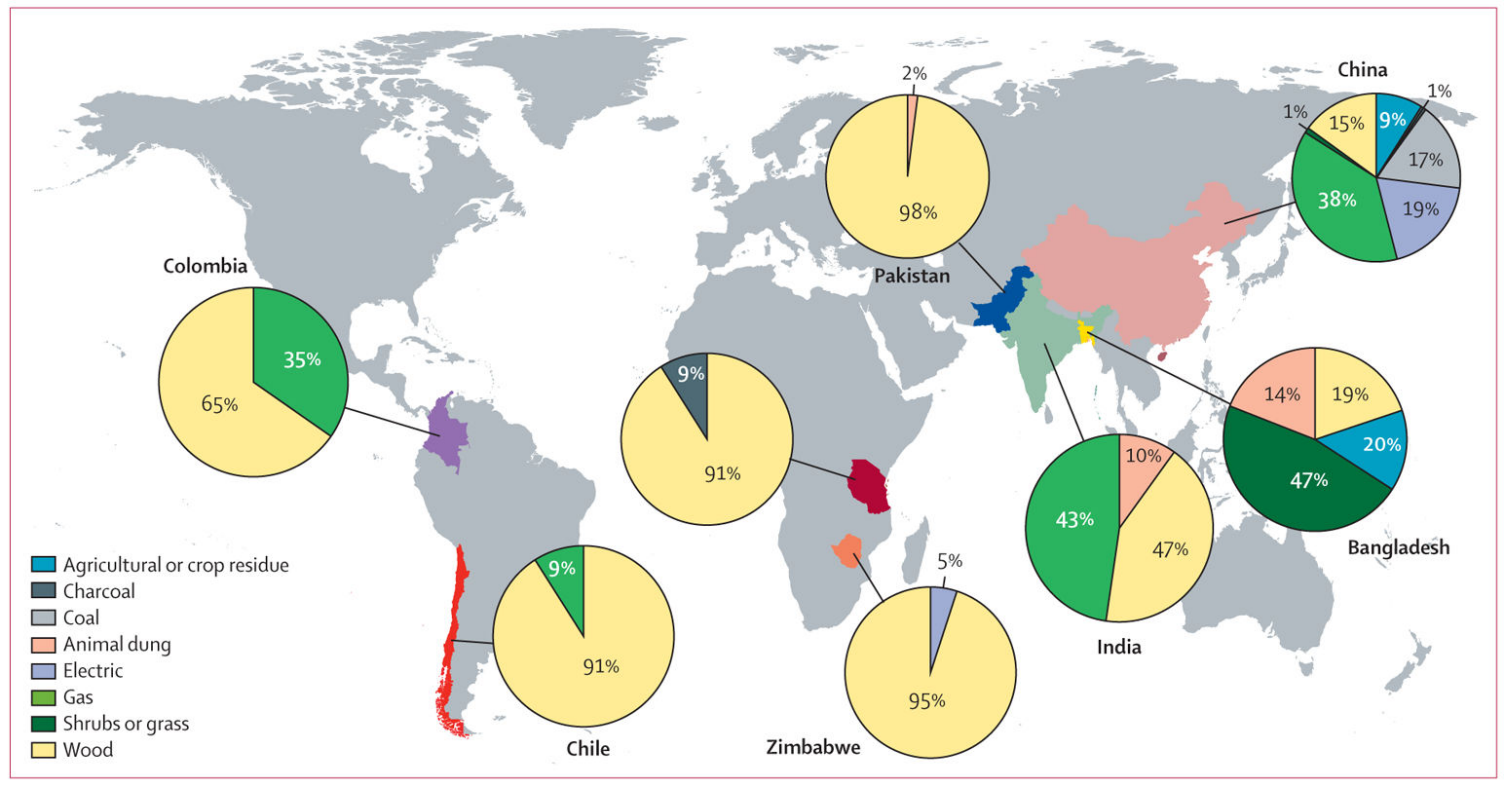
Primary fuel proportions sampled from each country in the PURE-AIR study

Polluting primary cooking fuels were used by 1436 (57%) households. Wood was the most prevalent primary cooking fuel in African, south Asian, and South American countries (figure 1). Open fires were most commonly used in Pakistan, Tanzania, Zimbabwe, and Colombia; mud stoves were most frequently used in India and Bangladesh; and manufactured chimney stoves were most prevalent in China and Chile. Fuel stacking (use of multiple fuels to meet cooking needs) occurred in 981 (39%) PURE-AIR households; the prevalence of stacking varied greatly, ranging from 1% (one of 132 households) in Karachi, Pakistan, to 88% (111 of 126 households) in Jiangsu, China (appendix p 5). Overall, 98% of households stacking fuels were in China, India, Colombia, and Chile; in India, the prevalence of stove stacking among PURE-AIR communities during 48 h monitoring (444 [55%] of 811 households) was around 20% higher than that of China (465 [37%] of 1244), Colombia (30 [39%] of 77), and Chile (27 [36%] of 75). 207 (24%) of869 households using gas as a primary fuel cooked with a polluting secondary fuel during the 48 h monitoring period. Participants using animal dung or shrubs or grass as primary fuels more frequently cooked outdoors, whereas participants using other primary fuels more commonly cooked indoors (table 1).

Self-reported average cooking time (primary fuel only) was approximately 2·3 h per day (table 1). Average daily cooking time was 0·7–1·1 h shorter among gas users (2·0 h per day) and electric stove users (1·6 h per day) than among wood stove users (2·7 h per day). Participants using animal dung cooked the longest, on average (4·8 h per day). 644 (25%) of 2541 households were heated with polluting fuels in open fires (299 [12%]), mud stoves (263 [10%]), or chimney stoves (82 [3%]) during the 48 h monitoring period.

998 personal samples (556 from female participants and 442 from male participants) were collected concurrently with kitchen monitoring. The average participant age was 60 years (range 38–84). On average, women spent almost three times as many hours per day in the kitchen as men (1·9 h versus 0·7 h; appendix p 12). 262 (47%) of 556 female participants reported their occupation as homemaker, compared with 44 (10%) male participants, and approximately a third of male participants (n=138) and female participants (n=139) self-reported exposure to “specific air pollution sources (eg, fires, industrial processes, traffic) at work” during the monitoring period (appendix p 12); we considered these participants as having occupational air pollution exposures. 172 (39%) male participants smoked tobacco products during monitoring. Although only 13 (2%) female participants smoked, 195 (35%) reported exposure to second-hand smoke during the 48 h monitoring period.

**Figure 2:**
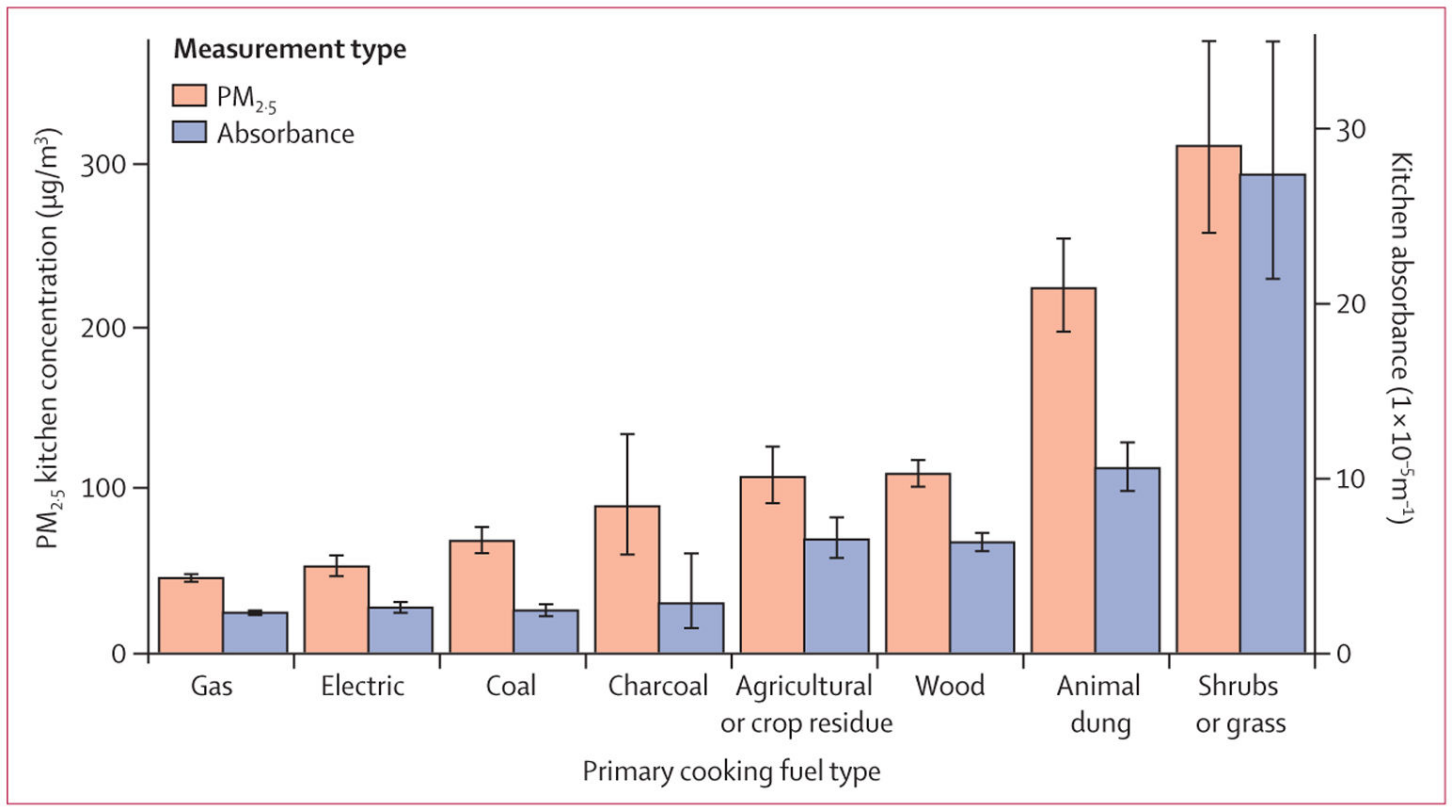
Summary of PM_2·5_ kitchen concentrations (μg/m^3^) and absorbance levels (1×10^−5^m^−1^) by primary fuel type Error bars are 95% CIs. Point estimates are geometric means.

Average 48 h household PM_2·5_ kitchen concentrations in households using wood as a primary cooking fuel (109 μg/m^3^ [95% CI 102–118]) were twice as high as concentrations from households using gas (45 μg/m^3^ [43–48]) or electric (53 μg/m^3^ [47–60]) cooking fuels (figure 2). Average PM_2·5_ concentrations from the most polluting fuels were higher than those from gas and electric fuels (animal dung, four times higher: 224 μg/m^3^ [95% CI 197–254]; shrubs or grass, five times higher: 276 μg/m^3^ [223–342]). Longer self-reported average daily cooking times were associated with increasing average PM_2·5_ kitchen concentrations in a dose-response manner among all polluting fuel types (table 2). 1915 (75%) of 2541 kitchen PM_2·5_ measurements, including 694 (63%) of 1105 measurements within households using clean fuels, were above the WHO Interim Target-1 (35 μg/m^3^ annual average).

Average PM_2·5_ kitchen concentrations remained substantially higher in households cooking with wood than in those using gas when stratifying by season (summer or winter) in PURE-AIR subnational regions where sampling spanned both seasons (appendix p 21). Seasonal differences in PM_2·5_ concentrations in some PURE-AIR subnational regions were likely to be partly due to household heating; heating via polluting fuels in mud stoves or open fires substantially increased average 48 h PM_2·5_ kitchen concentrations in the winter compared with summer among households primarily cooking with gas in Chennai, India (53 μg/m^3^ [95% CI 47–59] *vs* 32 μg/m^3^ [26–38]) and Liaoning, China (152 μg/m^3^ [70–330] *vs* 39 μg/m^3^ [29–52]; appendix p 21).

Black carbon and PM_2·5_ kitchen concentrations were highly correlated (*r*=0·88; p<0·0001); an increasing black carbon kitchen level gradient among polluting primary fuel types was also observed (figure 2). The average absorbance among households using clean primary fuels was less than half that of households using biomass primary fuel types (except for charcoal). However, minimal differences in black carbon concentrations existed between households using gas or electricity and coal or charcoal as primary fuels, despite a nearly two-fold variation in PM_2·5_ concentrations.

There was considerable between-country variation in household PM_2·5_ concentrations (intra-class correlation [ICC]_country_=0·61) and black carbon absorbance (ICC_country_=0·59) within the same primary cooking fuel type (appendix p 31). For example, among households cooking with wood, average PM_2·5_ concentrations from chimney stoves in China (50 μg/m^3^ [95% CI 45–55]) were half as high as those from mud stoves used in India (105 μg/m^3^ [96–116]). Average PM_2·5_ concentrations in households cooking with wood open fires in Bangladesh and Pakistan (383 μg/m^3^ [95% CI 339–435]) and African countries (318 μg/m^3^ [266–381]) were approximately three to four times higher than in households using mud stoves in India. Average PM_2·5_ concentrations in households using gas fuels in South America (20 μg/m^3^ [95% CI 17–23]) were half as high as in households using gas fuels in China (46 μg/m^3^ [43–49]) and India (50 μg/m^3^ [46–54]; table 2). Similarly, average black carbon kitchen concentrations in households cooking with wood in South America (2·1×10^−5^m^−1^ [95% CI 1·7–2·6]) and China (3·1×10^−5^m^−1^ [2·8–3·5]) were 33–50% lower than in households using wood in India (6·6×10^−5^m^−1^ [5·9–7·4]). Average black carbon concentrations in households cooking with wood in Africa (13·3×10^−5^m^−1^ [95% CI 11·1–15·8]) and in Pakistan and Bangladesh (25·0×10^−5^m^−1^ [21·6–28·8]) were two to four times higher than in households cooking with wood in India (appendix p 25). Thus, among households primarily cooking with wood, a ten-fold variation existed between countries in average 48 h measurements of PM_2·5_ (95% CI 40–380 μg/m^3^; table 2) and black carbon (2·1–25·0×10^−5^m^−1^; appendix p 25). A similar country-level pattern in average kitchen absorbance levels existed among households using gas fuels; black carbon levels in China (2·1×10^−5^m^−1^ [95% CI 2·0–2·3]) and India (2·7×10^−5^m^−1^ [2·5–3·0]) were twice as high as in South American countries (1·1×10^−5^m^−1^ [0·9–1·3]).

Among households using wood as a primary cooking fuel, use of gas as a secondary cooking fuel resulted in nearly 50% lower average PM_2·5_ concentrations (78 μg/m^3^ [95% CI 70–87]; table 2) and 50% lower average black carbon kitchen concentrations (4·3×10^−5^m^−1^ [95% CI 3·8–4·9]; appendix p 25) than use of only wood for cooking (146 μg/m^3^ [132–162] and 8·3×10^−5^m^−1^ [7·5–9·3]). Using animal dung as a secondary fuel with gas as a primary fuel was associated with approximately three times higher average PM_2·5_ concentrations (142 μg/m^3^ [95% CI 96–211]) and black carbon concentrations (6·5×10^−5^m^−1^ [95% CI 4·5–9·3]) than using only gas for cooking (44 μg/m^3^ [42–48] and 2·1×10^−5^m^−1^ [1·9–2·3]; table 2; appendix p 25).

No significant difference was observed between average 48 h personal PM_2·5_ exposures between female (67 μg/m^3^ [95% CI 62–72]) and male (62 μg/m^3^ [58–67]) participants. This finding held at a country level, except among PURE communities in Bangladesh and Pakistan, where female PM_2·5_ and black carbon exposures were significantly higher than male exposures (table 3; appendix p 26). In PURE communities within China and South American countries, average female PM_2·5_ exposures were 2–8 μg/m^3^ lower than male exposures (table 3).

**Figure 3:**
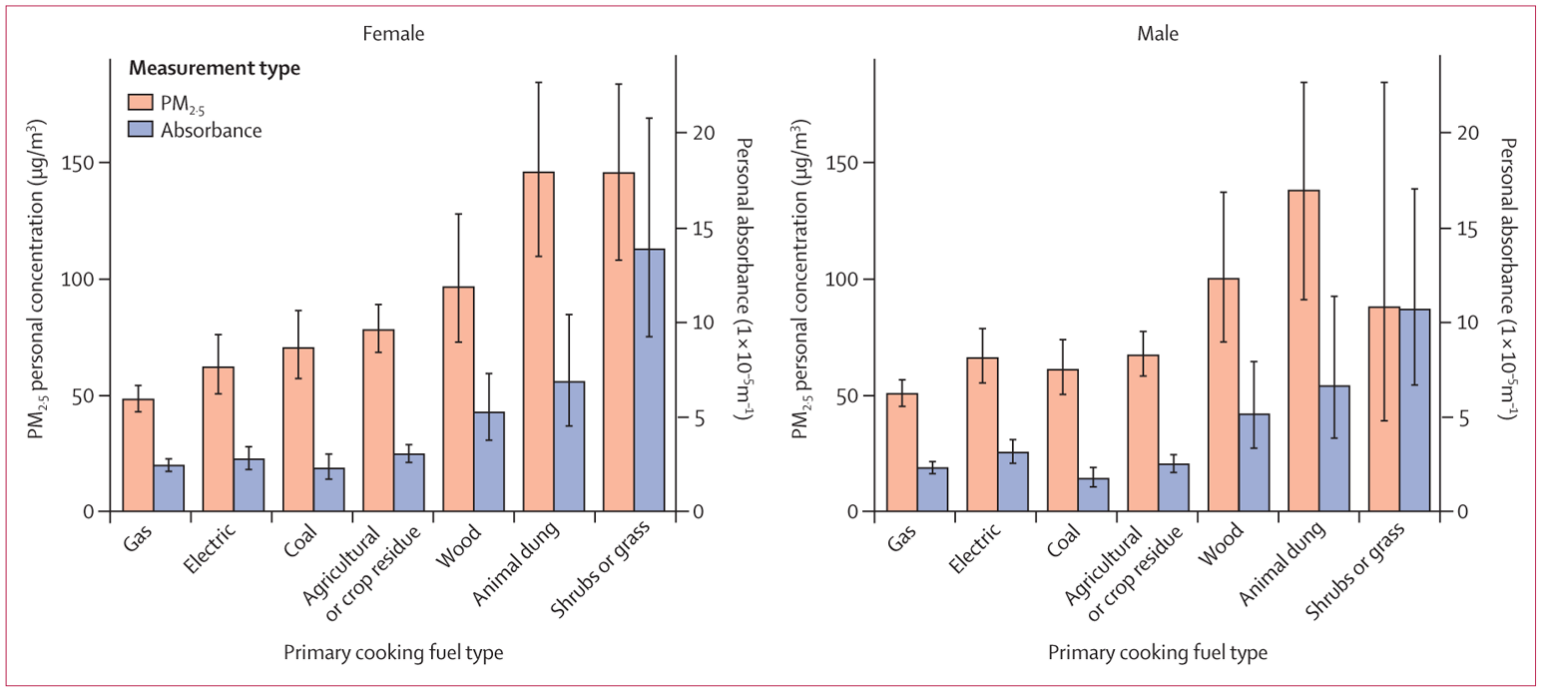
Summary of PM_2·5_ personal exposures (μg/m^3^) and absorbance levels (1×10^−5^m^−1^) by sex and primary fuel type Error bars are 95% CIs. Point estimates are geometric means.

Female participants cooking with gas as a primary fuel had 30 μg/m^3^ lower average PM_2·5_ exposures than female participants using wood as a primary fuel (48 μg/m^3^ [95% CI 43–54] *vs* 78 μg/m^3^ [68–89]; figure 3). Although average black carbon exposures were generally lower among participants using clean fuels than among those using polluting fuels, male participants living in households cooking with wood as a primary fuel had slightly lower average black carbon exposures than did those living in households primarily using electric stoves (figure 3).

Behavioural factors substantially affected personal exposure measurements. Average 48 h PM_2·5_ concentrations of both men and women were approximately 20 μg/m^3^ higher among those exposed to air pollution sources during work than in those reporting no occupational exposure (table 3). Average male and female black carbon exposure concentrations did not differ significantly between those reporting exposure and those reporting no exposure to occupational air pollution sources (appendix p 26). Younger participants (aged 43–60 years) had higher PM_2·5_ and black carbon exposures than older participants (aged 61–84 years). Male participants smoking tobacco products during the 48 h monitoring period had marginally higher (12 μg/m^3^) average PM_2·5_ exposures than male participants who did not smoke. Male and female participants who reported exposure to second-hand smoke (regardless of smoking status) had substantially higher (approximately 20 μg/m^3^) average PM_2·5_ and black carbon exposures than male and female participants who did not have exposure to second-hand smoke.

Mean male-to-kitchen and female-to-kitchen ratios from 227 households with paired male–female samples (n=454) were nearly equivalent for PM_2·5_ (0·79 [95% CI 0·71–0·88]) and 0·82 [0·74–0·91]) and black carbon (0·64 [0·45–0·92] and 0·68 [0·46–1·02]; appendix p 19). Female-to-kitchen and male-to-kitchen PM_2·5_ and black carbon exposure ratios were near or above 1 for most primary fuels (except for wood and shrubs or grass; range 0·4–0·7). The median male-to-female exposure ratio was 1·0 for both PM_2·5_ and black carbon (range 0·9–1·1) across all primary fuel types. However, at a country level, male-to-female PM_2·5_ ratios were greater than male-to- female ratios for black carbon in Chile, Colombia, and Pakistan; the reverse was true in China and India (appendix p 19).

Personal exposures were moderately correlated with kitchen PM_2·5_ concentrations (*r*=0·69; p<0·0001) and black carbon absorbance (*r*=0·63; p<0·0001; appendix p 30). When stratifying by sex, the correlation between female exposures and kitchen concentrations was higher than that of male exposures for both PM_2·5_ (*r*=0·71 [p<0·0001] *vs r*=0·65 [p<0·0001]) and black carbon (*r*=0·67 [p<0·0001] *vs r*=0·57 [p<0·0001]). The correlation between average black carbon and PM_2·5_ kitchen concentrations and personal exposures was modified by kitchen type in a monotonically decreasing manner (eg, among PM_2·5_ kitchen concentrations and female exposures: *r*=0·80 [p<0·0001] in single-room indoor kitchens, *r*=0·66 [p<0·0001] in multi-room indoor kitchens and *r*=0·46 [p<0·0001] in outdoor kitchens; appendix p 23). A sensitivity analysis examining PM_2·5_ exposures by UPAS wearing location (armband or harness) revealed no significant differences in exposures (appendix p 14).

## Discussion

The PURE-AIR study included PM_2·5_ and black carbon measurements related to household air pollution for 2541 households and 998 individuals in 120 diverse, rural communities within eight countries. Clear gradients in PM_2·5_ and black carbon kitchen concentrations were observed across primary cooking fuels; households using clean primary fuels had approximately two to five times lower average PM_2·5_ and black carbon kitchen concentrations than households using polluting primary fuels. Fuel stacking occurred in 981 (39%) households, and using clean secondary fuels was associated with 50% lower PM_2·5_ and black carbon concentrations. The use of clean primary cooking fuels also resulted in lower personal PM_2·5_ and black carbon exposures than the use of polluting fuels. Participants using gas as a primary fuel cooked for an average of 0·7 h per day less than participants using wood, suggesting that gas stoves can offer cumulative time savings.^23,24^

Stove characteristics and secondary fuel type affected measured PM_2·5_ and black carbon concentrations; among countries using different wood stoves (eg, chimney stoves in China, mud stoves in India, and open fires in Bangladesh, Pakistan, and African countries), there was a ten-fold variation in average PM_2·5_ kitchen concentrations (approximately 40–380 μg/m^3^; table 3) and black carbon absorbance (2·1–25·0×10^−5^m^−1^; appendix p 25). This analysis showed that using polluting secondary cooking fuels (eg, animal dung) in conjunction with gas as a primary fuel could potentially increase average 48 h PM_2·5_ and black carbon kitchen levels by 300%, from 44 μg/m^3^ to 142 μg/m^3^ (table 3) and from 2·1×10^−5^m^−1^ to 6·5×10^−5^m^−1^ (appendix p 25). Conversely, using a clean secondary fuel with a primary wood stove could decrease PM_2·5_ and black carbon kitchen concentrations by 50%. Accounting for fuel stacking and stove type in addition to primary cooking fuel type in household air pollution risk assessments is therefore important for reducing potential PM_2·5_ exposure misclassification.^25^

Despite female participants spending an average of 1·2 h per day longer in the kitchen than male participants (appendix p 12), median PM_2·5_ and black carbon personal-to-kitchen exposure ratios were identical for male and female participants (0·89 *vs* 0·86). The PM_2·5_ ratio in the PURE-AIR study is higher than previous median PM_2·5_ personal to kitchen ratios (0·74 for women *vs* 0·45 for men)^8,26^ used in GBD 2017.^7^ Higher median PM_2·5_ and black carbon personal-to-kitchen ratios in the PURE-AIR study were driven by PURE communities in four countries (China, India, Chile, and Columbia) where personal-to-kitchen ratios were generally higher than 0·9 (appendix p 19). In the four other countries (Bangladesh, Pakistan, Tanzania, and Zimbabwe), median PM_2·5_ and black carbon personal-to-kitchen ratios in PURE communities were lower than 0·5.

Greater homogeneity among black carbon and PM_2·5_ exposures between sexes among PURE communities in some countries is probably not attributable to increased smoking rates among male participants, as minimal differences existed in average PM_2·5_ concentrations among male and female non-smokers in households using gas as a primary fuel. Minor differences in average PM_2·5_ exposures by sex deviate from findings of previous household air pollution studies; in GBD 2017 and the HAPIT,^7,12^ a male-to-female exposure ratio of 0·6 is the default,^8^ whereas the median PM_2·5_ male-to-female exposure ratio in PURE-AIR was 1·0. PM_2·5_ and black carbon concentrations for one sex could serve as a viable household air pollution exposure proxy for the other in some settings. The health burden related to household air pollution in men might also be underestimated when assuming average male PM_2·5_ and black carbon exposures are consistently lower than female exposures across all low-income and middle-income countries. From the perspective of PM_2·5_ and black carbon exposures, these findings can have substantial global health implications by extending the framing of household air pollution beyond an issue primarily affecting women who are usually the primary household cook.

Across all polluting primary fuels, slightly higher PM_2·5_ personal-to-kitchen exposure ratios compared to black carbon exposure ratios (appendix p 19) suggest that sources other than biomass combustion probably contributed to PM_2·5_ exposures. The potential contribution of ambient pollution to PM_2·5_ exposures is further demonstrated by an increase of approximately 20 μg/m^3^ in average PM_2·5_ exposures among male and female participants reporting exposure to air pollution sources during work compared to participants who did not (table 3), with minimal differences in black carbon concentrations between the two groups (appendix p 26).

The relationship between PM_2·5_ and black carbon kitchen concentrations varied between countries. PURE-AIR communities in which polluting fuel combustion probably had the largest contribution to overall concentrations (kitchens with the highest black carbon fraction of PM_2·5_) included those in northern India, Pakistan, and Bangladesh (appendix p 28). Outdoor kitchens had a higher black carbon fraction of PM_2·5_ than indoor kitchens in Tanzania and two regions in India (appendix p 29), and the average kitchen absorbance levels from gas fuels in China (2·1×10^−5^m^−1^ [95% CI 2·0–2·3]) and India (2·7×10^−5^m^−1^ [2·5–3·0]) were twice as high as those from gas fuels in South American countries (1·1×10^−5^m^−1^ [0·9–1·3]; appendix p 25), possibly due to ambient sources of black carbon such as agricultural field burning. Furthermore, black carbon female-to-kitchen ratios among those using electric or gas stoves were higher than PM_2·5_ female-to-kitchen ratios in China, implying that ambient black carbon sources affected exposures. China accounts for the highest crop straw production globally,^27^ and around 25% of crop residue in India was burned in agricultural fields in 2017.^28^ Average male black carbon exposures from households in which coal and wood were the primary cooking fuels were lower than average male black carbon exposures from households where electric stoves were primarily used, which do not emit black carbon (appendix p 26), indicating male exposure to other black carbon sources, especially in India and China.

Average PM_2·5_ concentrations and exposures were above the WHO Interim Target-1 (35 μg/m^3^ annual average) across all primary fuel types, including clean fuels. Kitchen concentrations from gas and electric stoves were two to four times higher in some western Chinese provinces (Liaoning and Shaanxi) than in eastern Chinese provinces (Jiangsu; appendix p 8), suggesting high ambient air pollution levels in China. Ambient air pollution might be partly driven by community-level use of polluting fuels^29^ as biomass stove emissions can disperse and infiltrate neighbouring homes.^30^ Therefore, meeting WHO Air Quality Guidelines will require community-level transition to clean cooking fuels, and potentially emission reductions from other ambient pollution sources.^31^

The measured PM_2·5_ concentrations associated with each primary fuel type were considerably lower than estimates from a global PM_2·5_ modelling study based on the WHO global household air pollution database, where modelled concentrations were as follows: 104 μg/m^3^ (95% CI 39–273) for gas and electricity, 319 μg/m^3^ (119–838) for coal, and 958 μg/m^3^ (359–2520) for animal dung.^11^ Substantially lower PURE-AIR measurements might result from inclusion of studies done before 2000 in the WHO global household air pollution database, when household air pollution levels were likely to be higher in many low-income and middle-income countries, and also the demography of PURE households, which generally had a less than 1 h commute to research laboratories and might represent less rural communities with higher socioeconomic levels than communities sampled in previous household air pollution studies. As PURE-AIR included communities originally recruited for a study not focused on household air pollution, the findings might be more representative of rural exposures than studies focused on household air pollution that generally selectively recruit from communities with a high prevalence of household air pollution. These recent measurements might also represent broader trends in lower exposures due to increasing use of cleaner cooking fuels^16^ or reductions in family size, or both.

The PURE-AIR study leveraged the research capacity of the multinational PURE study, remote field-staff training, easy to use air samplers, real-time quality control measures, and a rapid, low-cost image-based reflectance method (proxy for black carbon concentrations) to enable scale up of PM_2·5_ and black carbon absorbance measurements to 120 communities in eight countries in a 2-year period. All PURE-AIR monitoring followed a harmonised protocol, minimising potential biases associated with pooling measurements across studies with different designs, measurement periods, monitoring equipment, and analytical methods. Although laboratory testing indicated a small coefficient of variation (5%) among duplicate UPAS measurements,^18^ a non-negligible difference in kitchen concentrations (8·5 μg/m^3^) among collocated UPAS monitors warrants further field testing, although this was possibly due to low sample sizes and poorly mixed kitchen environments. Wearing compliance of the UPAS during 48 h personal sampling was not included in this analysis (and is not commonly reported in the literature). GPS recorded by the UPAS revealed that 45 (5%) participants did not spend time away from their household during 48 h sampling (appendix p 34), which potentially signals high compliance with personal monitoring.

The PURE-AIR study was restricted to rural PURE communities with more than 10% polluting fuel use at baseline; the communities are not nationally representative of rural populations in each country. Given the pace of urbanisation during the 10–15-year follow-up period, some communities defined as rural according to baseline criteria might now be considered peri-urban.^16^ As we were not able to collect information on participant refusals, personal measurements might not be representative of PURE-AIR participants within each community.

Although 48 h monitoring is less sensitive to individual cooking events than a 24 h monitoring period, it might not represent longer-term exposures. Although repeat seasonal measurements were not done in all PURE-AIR communities because of logistical constraints, repeat seasonal measurements in 26 households in India and China, as well as a sensitivity analysis within eight PURE-AIR subnational regions (appendix p 21), revealed increases in kitchen concentrations in winter months compared to summer months in several countries (India, China, and Chile) with gas and wood as primary cooking fuels. As such, PURE-AIR measurements might not reflect annual average levels in some locations, but do provide multinational data on the range of concentrations by cooking fuel types.

PURE-AIR surveys did not include questions about polluting fuels used for lighting (eg, kerosene), which might have an important role in household air pollution, especially black carbon. Analysis of household heating was restricted as most households in each community did not heat their homes or used similar heating methods during the sampling period. However, among households in one subnational region in India and China, cooking with gas but using wood for heating (cooking in mud stoves in India and open fires in China), a significant increase in average kitchen concentrations relative to households with no heating was detected. Because of logistical constraints, outdoor air pollution concentrations were not monitored.

In conclusion, the PURE-AIR study illustrates potential global health and climate co-benefits of using clean cooking fuels, through reduced PM_2·5_ and black carbon concentrations. Although using clean primary fuels substantially lowered PM_2·5_ kitchen concentrations, 75% of all kitchen measurements, including 63% among households using clean fuels, were above the WHO Interim Target-1, suggesting that mitigation of ambient air pollution sources is needed to maximise the benefits to health and the climate. PURE-AIR measurements can be informative to global health stakeholders interested in characterising the health and climate impacts of household air pollution in future risk assessments.

## Supplementary Material

1

## Figures and Tables

**Table 1:** Characteristics of households included in the PURE-AIR study by primary cooking fuel type

	All households	Gas	Electric	Coal	Charcoal	Agricultural or crop residue	Wood	Animal dung	Shrubs or grass
Households (%)	2541	869 (34%)	236 (9%)	209 (8%)	8 (0%)	144 (6%)	903 (36%)	103 (4%)	69 (3%)
Country or region (%)									
China	1244 (49%)	478 (55%)	232 (98%)	208 (99%)	6 (75%)	117 (81%)	191 (21%)	2 (2%)	10 (14%)
India	811 (32%)	342 (40%)	1 (1%)	1 (1%)	0	2 (1%)	383 (42%)	80 (78%)	0
Other south Asia (Bangladesh and Pakistan)	258 (10%)	1 (0%)	0	0	0	25 (17%)	152 (17%)	21 (20%)	59 (86%)
South America (Chile and Colombia)	152 (6%)	47 (5%)	0	0	0	0	105 (12%)	0	0
Africa (Tanzania and Zimbabwe)	78 (3%)	1 (0%)	3 (1%)	0	2 (25%)	0	72 (8%)	0	0
Fuel stacking (%)	981 (39%)	338 (39%)	60 (25%)	38 (18%)	0	83 (58%)	375 (42%)	79 (77%)	8 (12%)
Secondary fuel (%)									
None	1570 (61%)	523 (60%)	183 (78%)	175 (84%)	8 (100%)	62 (43%)	528 (59%)	24 (23%)	61 (88%)
Gas	409 (16%)	..	27 (11%)	0	0	20 (14%)	283 (31%)	75 (73%)	2 (3%)
Electric	314 (12%)	139 (16%)	..	33 (16%)	0	54 (38%)	77 (9%)	0	6 (9%)
Coal	17 (1%)	3 (1%)	7 (3%)	..	0	3 (2%)	2 (0%)	0	0
Charcoal	0	2 (0%)	0	0	..	1 (1%)	1 (0%)	0	0
Agricultural or crop residue	23 (1%)	17 (2%)	2 (1%)	0	0	..	2 (0%)	1 (1%)	0
Wood	198 (8%)	177 (20%)	17 (7%)	0	0	1 (1%)	..	2 (2%)	0
Animal dung	14 (1%)	8 (1%)	0	0	0	0	6 (1%)	..	0
Shrubs or grass	0	0	0	0	0	0	3 (0%)	1 (1%)	..
Kitchen type (%)*									
Inside (no separate room)	118 (5%)	96 (11%)	1 (1%)	1 (1%)	0	1 (1%)	19 (2%)	0	0
Inside (separate room)	1882 (74%)	726 (84%)	227 (97%)	203 (97%)	7 (88%)	115 (80%)	526 (58%)	56 (54%)	22 (32%)
Porch or veranda	83 (4%)	12 (1%)	5 (2%)	4 (2%)	1 (12%)	5 (3%)	36 (4%)	14 (14%)	11 (16%)
Outside (open air)	433 (17%)	24 (3%)	0	0	0	21 (15%)	314 (35%)	33 (32%)	36 (52%)
Mean cooking time (primary fuel only; h per day)	2·3 (1·4)	2·0 (1·1)	1·6 (0·7)	1·4 (1·0)	1·7 (0·9)	1·9 (1·8)	2·7 (1·2)	4·8 (1·8)	2·3 (0·8)
Kitchen ventilation									
Chimney	842 (33%)	155 (18%)	115 (49%)	192 (92%)	5 (63%)	99 (69%)	211 (23%)	55 (53%)	10 (14%)
Window	1904 (75%)	785 (90%)	213 (90%)	190 (91%)	7 (88%)	114 (79%)	521 (58%)	56 (54%)	18 (26%)
Heating fuel type† (%)									
No heating	1692 (67%)	574 (66%)	152 (64%)	177 (85%)	5 (63%)	49 (34%)	567 (62%)	101 (98%)	67 (98%)
Electric or gas	195 (8%)	148 (17%)	31 (13%)	1 (0%)	0	9 (6%)	4 (1%)	1 (1%)	1 (1%)
Mud stove	261 (10%)	35 (4%)	23 (10%)	14 (7%)	0	76 (53%)	113 (13%)	0	1 (1%)
Open fire	300 (12%)	106 (12%)	26 (11%)	16 (8%)	3 (38%)	9 (6%)	138 (15%)	1 (1%)	0
Chimney stove	82 (3%)	3 (1%)	0	0	0	0	79 (9%)	0	0
Smoking in home (%)	708 (28%)	235 (27%)	99 (42%)	63 (30%)	2 (25%)	41 (28%)	193 (21%)	44 (43%)	31 (45%)
Household asset index‡ (%)									
Tertile 1 (lowest)	1322 (52%)	309 (36%)	154 (65%)	165 (79%)	5 (63%)	95 (66%)	536 (59%)	28 (27%)	27 (39%)
Tertile 2	815 (32%)	349 (40%)	64 (27%)	31 (15%)	2 (25%)	32 (22%)	269 (30%)	42 (41%)	24 (35%)
Tertile 3 (highest)	316 (12%)	180 (21%)	15 (6%)	12 (6%)	1 (12%)	13 (9%)	73 (8%)	8 (8%)	14 (20%)
Education level§ (%)									
None	607 (24%)	104 (12%)	20 (8%)	40 (19%)	1 (12%)	18 (13%)	348 (39%)	47 (46%)	29 (42%)
Primary	809 (32%)	240 (28%)	90 (38%)	107 (51%)	2 (25%)	25 (17%)	305 (34%)	24 (23%)	16 (23%)
Secondary	996 (39%)	466 (54%)	120 (51%)	54 (26%)	5 (63%)	89 (62%)	218 (24%)	24 (23%)	20 (29%)
Trade or university	82 (3%)	44 (5%)	1 (0%)	5 (2%)	0	6 (4%)	19 (2%)	4 (4%)	3 (4%)

Data are n (%) or mean (SD).

* Kitchen type is a derived variable that was coded to match groupings reported in the WHO harmonised survey for monitoring household energy use.^21^ Participants who reported cooking indoors and having at least two rooms in the home were categorised as cooking indoors “in a separate room”. Those reporting having one room in the home were categorised as indoor cooking with “no separate room”. Participants who reported cooking inside with their kitchen being “partially open to the outside” were categorised as cooking on a “porch or veranda”. Those who reported cooking outdoors were assumed to cook “in open air”. No questions were asked in PURE surveys about whether the indoor kitchen was attached or detached from the main household.

† Percentages for heating fuel type do not add up to 100% due to non-response (0%).

‡ Household asset index was ranked at a national level and grouped into country-stratified tertiles.^22^ Percentages for household asset index do not add up to 100% due to non-response (3%).

§ Highest education level in the household (baseline). Percentages for education level do not add up to 100% due to non-response (2%).

**Table 2:** Summary of average 48 h PM_2·5_ kitchen concentrations by primary fuel type

	Gas (n=869)	Electric (n=236)	Coal (n=209)	Charcoal (n=8)	Agricultural or crop residue (n=144)	Wood (n=903)	Animal dung (n=103)	Shrubs or grass (n=69)
**Kitchen PM_2·5_ (μg/m^3^)**								
Total	45 (43–48)	53 (47–60)	68 (61–77)	92 (58–146)	106 (91–125)	109 (102–118)	224 (197–254)	276 (223–342)
Country or region								
China	46 (43–49)	53 (47–60)	68 (61–77)	78 (48–127)	89 (74–106)	50 (45–55)	85 (40–182)	65 (43–100)
India	50 (46–54)	..	..	..	140 (17–1126)	105 (96–116)	209 (181–242)	..
Other south Asia (Bangladesh and Pakistan)	..	..	..	..	244 (200–298)	383 (339–435)	317 (259–388)	352 (296–420)
South America (Chile and Colombia)	20 (17–23)	..	..	..	..	41 (34–49)	..	..
Africa (Tanzania and Zimbabwe)	..	26 (14–47)	..	136 (126–147)	..	318 (266–381)	..	..
Secondary fuel								
None	44 (42–48)	54 (46–62)	71 (62–81)	92 (58–146)	122 (95–171)	146 (132–162)	287 (210–346)	324 (265–397)
Gas	..	70 (57–86)	..	..	70 (50–99)	78 (70–87)	206 (177–238)	210 (121–251)
Electric	45 (41–51)	..	56 (46–67)	..	102 (83–125)	46 (39–56)	..	62 (39–97)
Coal	139 (74–261)	47 (30–75)	..	..	134 (79–227)	..	..	..
Charcoal	191 (71–514)	..	..	..	..	..	..	..
Agricultural or crop waste	41 (31–53)	80 (73–87)	..	..	..	304 (200–463)	..	..
Wood	45 (40–50)	30 (22–38)	..	..	..	..	..	..
Animal dung	142 (96–211)	..	..	..	..	168 (111–256)	..	..
Shrubs or grass	..	..	..	..	..	284 (143–564)	..	..
**Cooking time during monitoring (primary fuel only; h per day)**								
0·0–1·0	47 (41–54)	70 (56–87)	69 (58–83)	65 (38–110)	104 (67–160)	76 (60–97)	..	162 (86–306)
1·1–2·0	44 (41–47)	48 (41–57)	69 (58–83)	93 (38–228)	94 (77–115)	97 (85–110)	266 (197–358)	225 (142–357)
2·1–3·0	47 (42–53)	53 (41–68)	78 (56–107)	136 (126–147)	181 (135–241)	101 (89–113)	245 (180–335)	311 (229–421)
≥3·1	48 (40–58)	98 (30–329)	51 (38–68)	..	188 (69–514)	150 (127–175)	219 (189–255)	372 (265–524)

Data are geometric means (95% CI).

**Table 3:** Summary of average 48 h PM_2·5_ personal exposures by primary fuel type

	All households		Gas		Electric		Coal		Agricultural or crop waste		Wood		Animal dung		Shrubs or grass	
	Male (n=442)	Female (n=556)	Male (n=l68)	Female (n=194)	Male (n=57)	Female (n=59)	Male (n=34)	Female (n=37)	Male (n=25)	Female (n=29)	Male (n=142)	Female (n=201)	Male (n=7)	Female (n=17)	Male (n=9)	Female (n=19)
Total	62 (58–67)	67 (62–72)	51 (45–56)	48 (43–54)	66 (55–78)	62 (50–76)	61 (52–78)	71 (57–86)	100 (73–138)	97 (73–128)	68 (59–78)	78 (69–89)	138 (91–210)	146 (110–194)	88 (39–199)	147 (109–197)
Country or region																
China	57 (52–62)	55 (50–61)	50 (43–59)	47 (38–56)	66 (55–78)	61 (49–75)	61 (52–78)	71 (58–88)	93 (64–136)	94 (68–129)	44 (37–54)	45 (36–54)	..	..	37 (3–405)	64 (32–128)
India	66 (57–77)	70 (62–80)	53 (45–63)	56 (48–64)	..	..	..	..	..	..	82 (64–107)	89 (74–114)	178 (132–240)	150 (105–216)	..	..
Other south Asia (Bangladesh and Pakistan)	103 (83–119)	158 (125–179)	..	..	..	..	..	..	147 (137–157)	148 (110–198)	90 (67–111)	148 (100–182)	73 (34–159)	147 (81–269)	135 (110–165)	183 (146–229)
South America (Chile and Colombia)	40 (30–51)	32 (25–38)	40 (28–53)	23 (18–28)	..	..	..	..	..	..	40 (25–64)	39 (28–50)	..	..	..	..
Africa (Tanzania and Zimbabwe)	114 (79–166)	146 (112–141)	..	..	..	85 (51–140)	..	..	..	..	120 (80–179)	153 (116–202)	..	..	..	..
Age, years																
43–60	71 (62–82)	79 (71–88)	61 (51–74)	52 (44–62)	60 (42–85)	65 (45–93)	57 (37–87)	76 (56–102)	100 (56–181)	122 (74–200)	83 (63–110)	86 (71–103)	131 (91–187)	159 (110–231)	153 (128–182)	190 (144–251)
61–84	57 (50–63)	54 (47–63)	47 (40–56)	49 (39–62)	82 (56–119)	55 (32–96)	63 (48–82)	58 (37–92)	100 (68–147)	92 (66–127)	62 (51–75)	65 (50–84)	184 (126–268)	119 (70–203)	67 (20–219)	94 (50–174)
Occupational air pollution exposure*																
Yes	75 (65–86)	82 (71–96)	63 (51–77)	53 (40–70)	81 (44–152)	62 (50–78)	50 (43–58)	71 (57–88)	100 (73–138)	97 (73–128)	78 (63–97)	96 (80–117)	236 (142–394)	95 (62–145)	132 (104–169)	148 (126–174)
No	57 (52–62)	63 (57–69)	46 (40–53)	47 (41–54)	63 (52–77)	56 (23–136)	64 (52–80)	..	..	..	58 (48–70)	66 (55–79)	112 (73–171)	175 (124–246)	53 (9–325)	146 (97–219)
Smoker																
Yes	70 (62–79)	91 (58–141)	63 (52–77)	74 (33–164)	72 (54–96)	..	75 (52–109)	..	104 (57–188)	..	68 (54–85)	105 (46–244)	138 (107–178)	..	164 (135–199)	..
No	58 (52–63)	67 (62–72)	44 (38–50)	47 (42–54)	58 (47–72)	62 (50–76)	61 (48–77)	71 (57–86)	98 (66–144)	97 (73–128)	67 (55–81)	78 (68–89)	138 (76–252)	146 (110–194)	74 (26–205)	147 (109–197)
Second-hand smoke exposure																
Yes	72 (64–81)	79 (70–90)	59 (48–71)	66 (52–83)	69 (50–93)	76 (55–105)	81 (55–121)	66 (48–91)	100 (60–164)	102 (64–164)	78 (63–96)	78 (111–168)	137 (110–165)	191 (118–197)	135 (110–165)	153 (118–197)
No	54 (49–60)	61 (55–67)	45 (39–51)	42 (36–48)	62 (51–76)	53 (40–70)	60 (47–76)	72 (55–93)	101 (71–144)	93 (65–134)	57 (46–71)	79 (66–94)	140 (48–407)	131 (91–188)	37 (3–405)	135 (53–346)

Data are geometric mean (95% CI) in units of μg/m^3^.

* Occupational air pollution represents participants who self-reported being exposed to specific air pollution sources (eg, fires, industrial processes, and traffic) while at work during the 48 h monitoring period.

## Research in context

### Evidence before this study

An estimated 2·8 billion people cooked with polluting fuels (eg, wood, coal, animal dung, and kerosene) in 2018. Evidence from household air pollution measurement studies demonstrates that cooking with polluting fuels is associated with higher concentrations of fine particulate matter (PM_2·5_) mass and black carbon (two important indicators of health and climate impacts), compared to clean cooking fuels (gas and electricity). An existing global modelling study that pooled PM_2·5_ kitchen and personal measurements (n=2208) from 44 published measurement studies available in the WHO global database of household air pollution measurements showed large variations in mean PM_2·5_ concentrations and female exposures across primary fuel types and geographical locations. These existing household air pollution measurement studies were generally done among small populations in few communities, and most individual monitoring data were collected from women who are more commonly the primary household cook than men. Compared to PM_2·5_, relatively little measurement data are available for household concentrations of black carbon. The magnitude of PM_2·5_ and black carbon household exposures therefore remains unclear.

### Added value of this study

The PURE-AIR study is among the largest and most diverse exposure assessments of PM_2·5_ and black carbon related to household air pollution, with measurements from 120 rural communities in eight countries (Bangladesh, Chile, China, Colombia, India, Pakistan, Tanzania, and Zimbabwe). The PURE-AIR study more than doubles the number of PM_2·5_ measurements available in the WHO global household air pollution database. By collecting information on both primary and secondary cooking fuels, the impact of multiple fuel combinations (ie, fuel stacking) on PM_2·5_ and black carbon kitchen concentrations was also assessed. Personal monitoring of both sexes in this study provides unique information about household air pollution exposures among men, who have often been considered to be at lower risk of PM_2·5_ and black carbon exposure from cooking than women. PURE-AIR measurements provide extensive information about the contribution of household cooking to overall exposures in different countries and the role of different cooking fuel types on emissions of air pollutants that contribute to global warming.

### Implications of all the available evidence

The PURE-AIR study provides important new information about variations in PM_2·5_ and black carbon kitchen concentrations and household air pollution exposures on a multinational scale. These measurements can be used to inform risk assessments and policy scenarios targeting household air pollution and can be integrated with health studies to further understand the relationship between exposure to household air pollution and adverse health effects.
