# Supplementary material for: Household and personal air pollution exposure measurements from 120 communities in eight countries: results from the PURE-AIR study
Source: Lancet Planet Health. Author manuscript; Available in PMC 2020 Oct 27. (PMC7591267; doi:10.1016/S2542-5196(20)30197-2)
Supplement: 1 [file NIHMS1635947-supplement-1.pdf]

THE LANCET
Planetary Health

Supplementary appendix

This appendix formed part of the original submission and has been peer reviewed. We post it as supplied by the authors.

Supplement to: Shupler M, Hystad P, Birch A, et al. Household and personal air pollution exposure measurements from 120 communities in eight countries: results from the PURE-AIR study. *Lancet Planet Health* 2020; **4:** e451–62.

Household and Personal Air Pollution Exposure Measurements from 120 Communities in 8 Countries: Results from the PURE-AIR Study

Matthew Shupler, MPH[1,2*]; Perry Hystad, PhD[3]; Aaron Birch, MSc[1]; Daniel Miller-Lionberg, MSc[4]; Matthew Jeronimo, BSc[1]; Raphael E. Arku, ScD[1,5]; Yen Li Chu, BSc[1]; Maha Mushtaha, BSc[6]; Laura Heenan, MSc[6]; Sumathy Rangarajan, MSc[6]; Pamela Seron, PhD[7]; Fernando Lanas, PhD[7]; Fairuz Cazor, BSc[7]; Prof. Patricio Lopez-Jaramillo, PhD[8]; Paul A. Camacho, MD[9]; Maritza Perez, MD[10]; Karen Yeates, MD[11,12]; Nicola West, RN[11]; Tatenda Ncube, BSc[13]; Brian Ncube, BSc[13]; Jephat Chifamba, PhD[13]; Rita Yusuf, PhD[14]; Afreen Khan, MHS[14]; Bo Hu, PhD[15]; Xiaoyun Liu, PhD[15]; Prof. Li Wei, PhD[15]; Lap Ah Tse, PhD[16]; Deepa Mohan, PhD[17]; Parthiban Kumar, BSc[17]; Prof. Rajeev Gupta, PhD[18]; Indu Mohan, MD[19]; KG Jayachitra, MSc[20]; Prem K. Mony, MD[20]; Kamala Rammohan, MD[21]; Sanjeev Nair, MD[21]; Lakshmi PVM, MD[22]; Vivek Sagar, PhD[22]; Rehman Khawaja, MA[23]; Romaina Iqbal, PhD[23]; Khawar Kazmi, MD[23]; Prof. Salim Yusuf, PhD[6]; Prof. Michael Brauer ScD[1]; on behalf of the *PURE-AIR Study*

1. School of Population and Public Health, University of British Columbia, Vancouver, British Columbia, Canada
2. Department of Public Health and Policy, University of Liverpool, Liverpool, United Kingdom
3. College of Public Health and Human Sciences, Oregon State University, Corvallis, Oregon, United States
4. Access Sensors Technologies, Fort Collins, Colorado, United States
5. School of Public Health and Health Sciences, University of Massachusetts Amherst, Amherst, Massachusetts
6. Population Health Research Institute, Hamilton Health Sciences, McMaster University, Hamilton, Ontario, Canada
7. Universidad de La Frontera, Temuco, Chile
8. Universidad de Santander (UDES), Bucaramanga, Colombia
9. FOSCAL, Floridablanca, Colombia
10. Universidad Militar Nueva Granada, Bogota, Colombia
11. Pamoja Tunaweza Research Centre, Moshi, Tanzania
12. Department of Medicine, Queen's University, Kingston, Ontario, Canada
13. Department of Physiology, University of Zimbabwe, Harare, Zimbabwe
14. School of Life Sciences, Independent University, Dhaka, Bangladesh
15. Medical Research & Biometrics Center, National Center for Cardiovascular Diseases, Fuwai Hospital, Chinese Academy of Medical Sciences
16. Jockey Club School of Public health and Primary Care, the Chinese University of Hong Kong, HKSAR, China
17. Madras Diabetes Research Foundation, Chennai, India
18. Eternal Heart Care Centre & Research Institute, Jaipur, India
19. Mahatma Gandhi Medical College, Jaipur, India
20. St. John's Medical College & Research Institute, Bangalore, India
21. Health Action By People, Thiruvananthapuram and Medical College, Trivandrum, India
22. Post Graduate Institute of Medical Education and Research, Chandigarh, India
23. Department of Community Health Science, Aga Khan University Hospital, Karachi, Pakistan

*Corresponding author: mshupler@mail.ubc.ca, m.shupler@liverpool.ac.uk, +1 604-827-2816

Years of Data Collection

Data collection for the PURE-AIR study occurred from 2017-2019, with monitoring equipment shipped directly between countries once sampling commenced in each respective community. As PURE baseline surveys were also conducted on a rolling basis, the time between PURE baseline and the PURE-AIR study varied by country (Table S1).

Table S1. Years of data collection for PURE baseline surveys and PURE-AIR monitoring.

	Year													
**Country**	**2004**	**2005**	**2006**	**2007**	**2008**	**2009**	**…**	**2012**	**2013**	**2014**	**…**	**2017**	**2018**	**2019**
Bangladesh					X								X	
Chile			X	X	X								X	X
China		X	X	X	X	X						X	X	X
Colombia		X	X	X	X	X								X
India	X	X	X	X								X	X	
Pakistan						X							X	X
Tanzania								X	X	X		X	X	X
Zimbabwe			X	X								X	X	

X PURE Baseline (red)

X PURE-AIR (blue)

Fuel Proportions by Country

In each PURE AIR country, stratified random sampling was used to select households proportional to baseline primary fuel percentages within each community. In most countries, polluting fuels were over-sampled to better capture the variation in $PM_{2.5}$ concentrations due to HAP. Due to a high amount of fuel switching between PURE baseline and follow up in India and China,[1] there were not enough households using polluting fuels at the time of PURE-AIR monitoring to fully match baseline fuel proportions (Table S2).

Table S2. Distribution of primary fuel types among PURE Baseline and PURE-AIR samples by country.

Country	Fuel Type	Baseline Proportion	PURE-AIR Proportion
India	Ag/crop	57 (1%)	2 (0%)
	Animal dung	464 (6%)	80 (10%)
	Charcoal	3 (0%)	1 (0%)
	Coal	66 (1%)	1 (0%)
	Electricity	7 (0%)	1 (0%)
	Gas	1475 (20%)	342 (42%)
	Kerosene	56 (1%)	2 (0%)
	Wood	5073 (70%)	383 (47%)
China	Ag/crop	2294 (20%)	117 (9%)
	Animal dung	151 (1%)	2 (0%)
	Charcoal	118 (1%)	6 (1%)
	Coal	3459 (30%)	208 (17%)
	Electricity	279 (2%)	232 (19%)
	Gas	2918 (25%)	478 (38%)
	Shrub/grass	807 (7%)	10 (1%)
	Wood	1313 (12%)	191 (15%)
Colombia	Ag/crop	20 (1%)	0
	Charcoal	24 (1%)	0
	Coal	175 (9%)	0
	Electricity	13 (1%)	0
	Gas	1045 (54%)	30 (40%)
	Wood	649 (34%)	47 (60%)
Chile	Gas	47 (13%)	17 (23%)
	Wood	307 (87%)	58 (77%)
Bangladesh	Ag/crop	162 (14%)	25 (20%)
	Animal dung	115 (13%)	18 (14%)
	Electricity	12 (1%)	0
	Gas	26 (3%)	0
	Shrub/grass	215 (29%)	59 (47%)
	Wood	340 (39%)	24 (19%)
Pakistan	Animal dung	31 (7%)	3 (2%)
	Gas	9 (2%)	1 (1%)
	Wood	369 (91%)	128 (97%)
Tanzania	Ag/crop	4 (1%)	0
	Charcoal	32 (6%)	2 (9%)
	Coal	10 (2%)	0
	Gas	2 (0%)	0
	Kerosene	88 (18%)	0
	Wood	359 (73%)	20 (91%)
Zimbabwe	Ag/crop	3 (1%)	0
	Animal dung	5 (1%)	0
	Charcoal	5 (1%)	0
	Electricity	14 (3%)	3 (6%)

	Gas	3 (1%)	1 (2%)
	Wood	423 (93%)	52 (92%)

Fuel Stacking

During the 48-hour monitoring, a wide variety of fuels were 'stacked' in households sampled in the PURE-AIR study; 11 different combinations were commonly reported (Figure S1). In India, the prevalence of fuel stacking among PURE-AIR communities was >20% in all five sub-national regions, while <20% of households in four (Beijing, Inner Mongolia, Jiangxi, Qinghai) of 11 sub-national in China regions stacked fuels during the monitoring period (Figure S2). In almost every case of stacking, a clean fuel was stacked with a polluting fuel, although some households in China stacked gas and electric fuels.

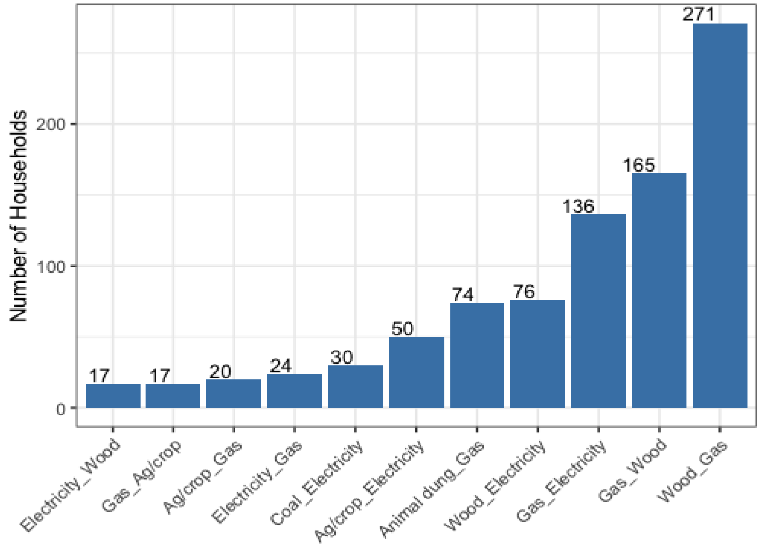

Figure S1. Prevalence of fuel stacking combinations among rural communities included in the PURE-AIR Study. The first fuel listed is the primary fuel and the second is the secondary fuel used.

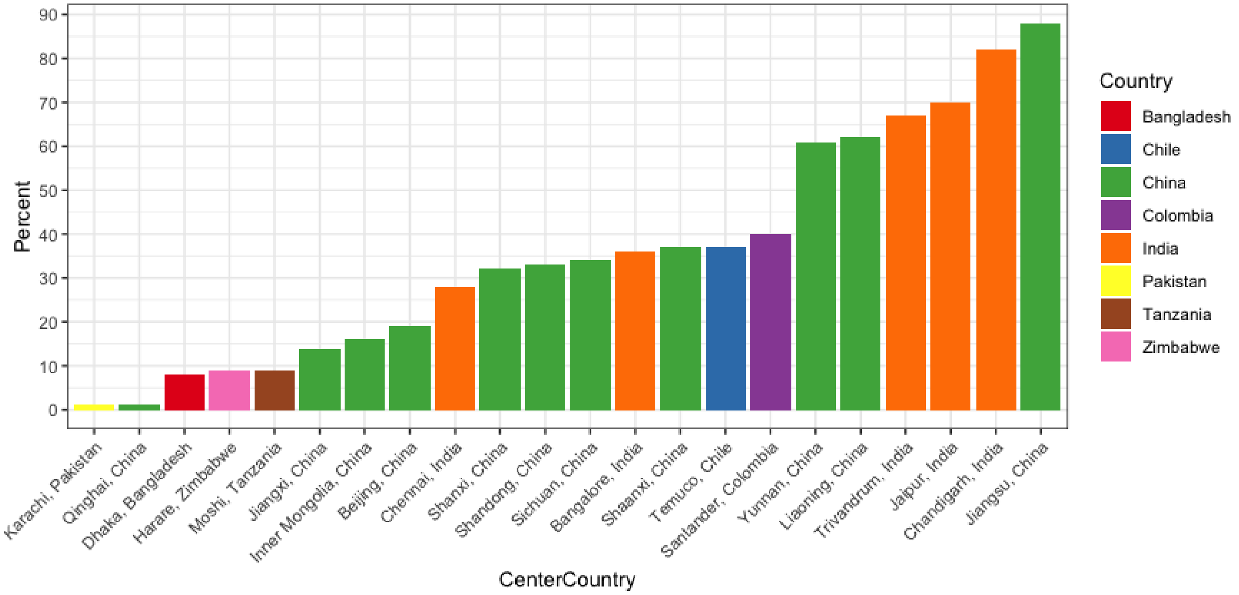

Figure S2. Prevalence of stacking in rural PURE-AIR communities by country and sub-national region.

In Jaipur, India, using gas as a secondary fuel with wood primary fuel during the 48-hour monitoring resulted in ~20 µg/m3 lower average $PM_{2.5}$ concentrations compared to households only cooking with wood (Figure S3). Further, using wood as a secondary fuel with gas primary fuel, as opposed to using only gas for cooking, led to increased average concentrations (~10 µg/m3) in Chennai.

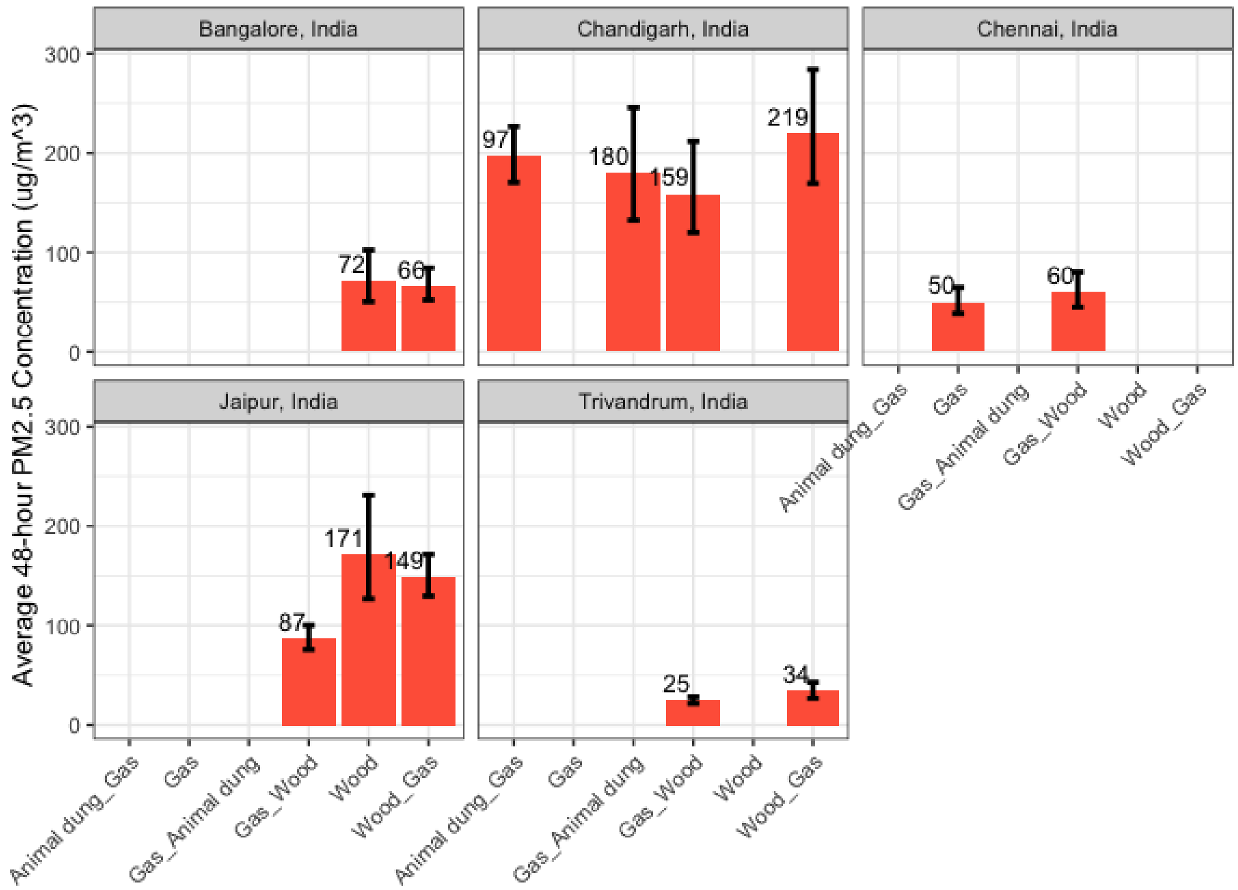

Figure S3. Average 48-hour $PM_{2.5}$ concentrations (95% CI) among primary and secondary fuel combinations among rural PURE-AIR communities in subnational-regions in India. The first fuel listed is the primary fuel and the second is the secondary fuel used. Fuel combinations with n<3 in a sub-national region were not included for brevity.

In China, more fuel stacking combinations existed during the 48-hour monitoring than in India. PURE-AIR households that used a clean secondary fuel with a polluting primary fuel during the monitoring period did not have significantly different concentrations than households using only polluting fuels. Lower average $PM_{2.5}$ kitchen concentrations among households using wood secondary fuels with electric primary fuels, compared to households using only gas or electric during the monitoring period (Figure S4), is likely due to regional differences in ambient air pollution. Fuel stacking of electricity and wood occurred in Yunnan, which had much lower average concentrations from gas and electric fuels, compared with Liaoning and Sichuan (Figure S5). Therefore, aggregating across all regions in China likely inflated the estimated kitchen $PM_{2.5}$ concentrations contributed by HAP from gas and electric stoves.

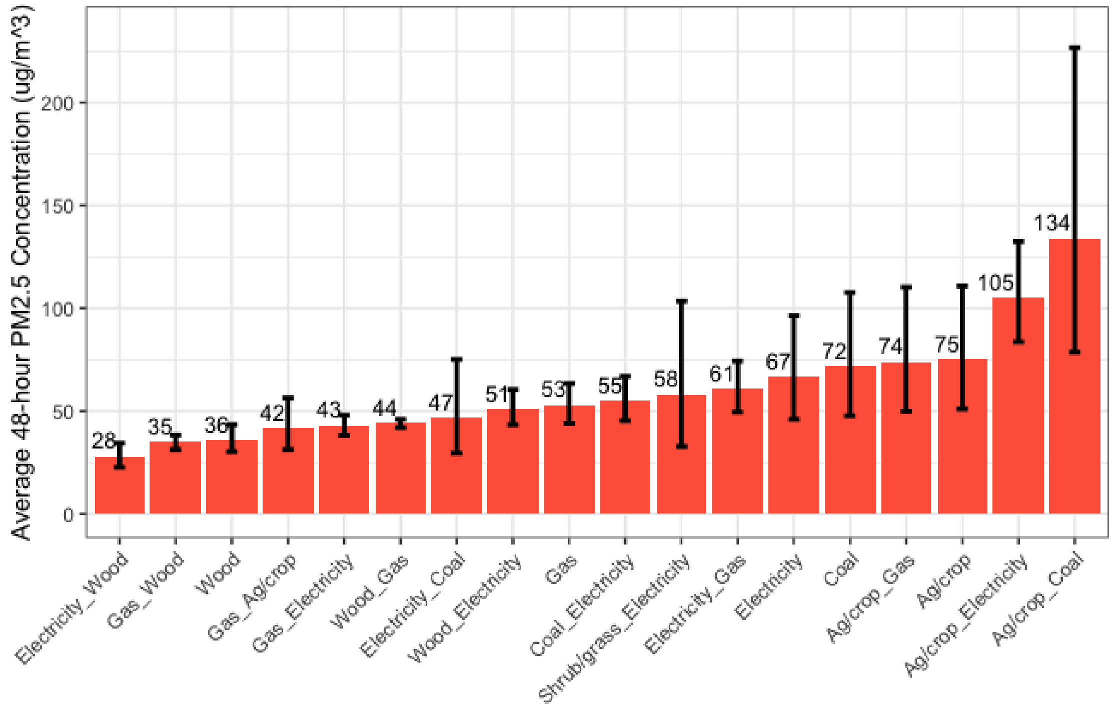

Figure S4. Average 48-hour $PM_{2.5}$ concentrations (95% CI) among primary and secondary fuel combinations in rural PURE-AIR communities in China. The first fuel listed is the primary fuel and the second is the secondary fuel used. Fuel combinations with n<3 were not included for brevity.

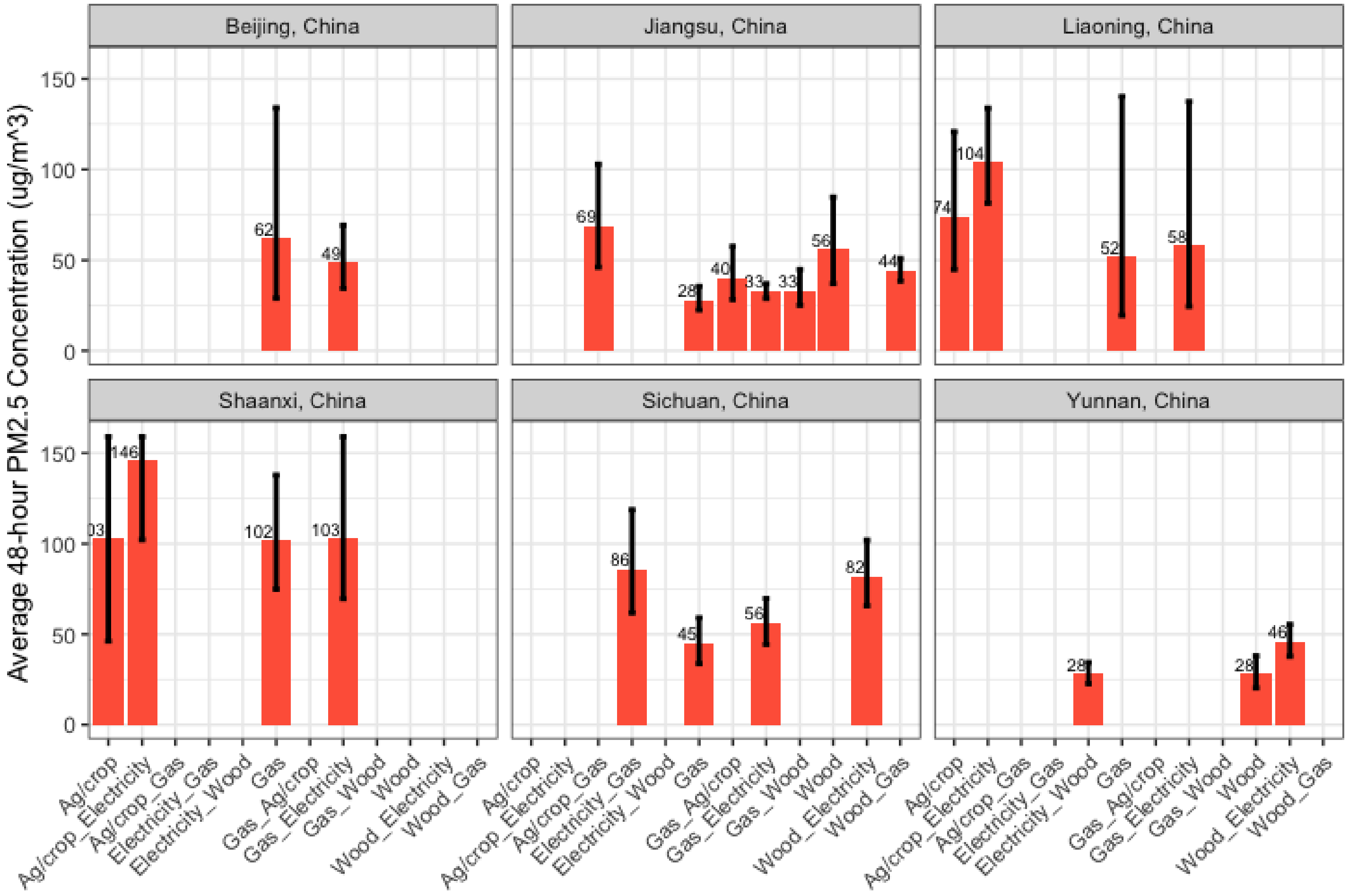

Figure S5. Average 48-hour $PM_{2.5}$ concentrations (95% CI) among primary and secondary fuel combinations among rural PURE-AIR communities in select sub-national regions in China in which fuel stacking was prevalent. The first fuel listed is the primary fuel and the second is the secondary fuel used. Fuel combinations with n<3 in a sub-national region were not included for brevity.

In Colombia, households using gas secondary fuel with wood had slightly lower (~15 μg/m^3) average 48-hour kitchen concentrations than households using only wood (Figure S6).

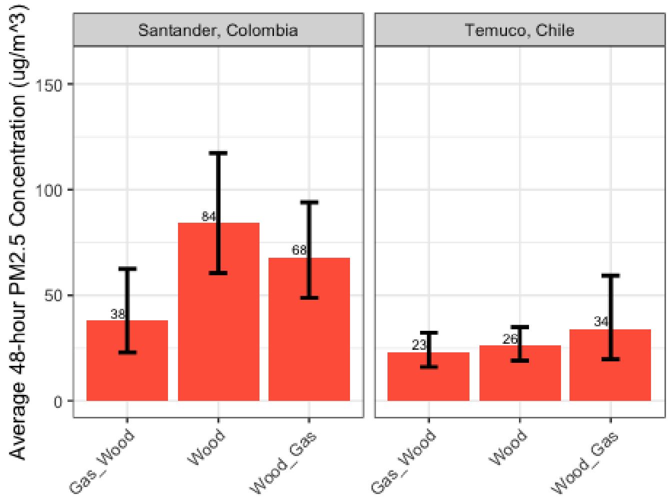

Figure S6. Average 48-hour HAP-$PM_{2.5}$ concentrations (95% CI) among primary and secondary fuel combinations among rural PURE-AIR communities in South American countries. The first fuel listed is the primary fuel and the second is the secondary fuel used.

Stove Technology

There were minimal differences observed in primary stove types used for cooking with polluting fuels within countries (Table S3). The most common stove types used in each PURE-AIR country were: chimney stoves (China, Chile), mud stoves (India, Bangladesh) and open fires (Colombia, Tanzania, Zimbabwe, Pakistan) (Figure S7).

Table S3. Distribution of most common primary stove types by PURE-AIR country and sub-national region. Primary stove types with n<3 in a sub-national region were not included for brevity.

Country	Sub-national region	Stove type	Primary fuel	Count (%)
Bangladesh	Dhaka	Mud	Ag/crop	25 (20%)
		Mud	Animal dung	18 (14%)
		Mud	Shrub/grass	59 (47%)
		Mud	Wood	24 (19%)
Chile	Temuco	Gas stove	Gas	17 (23%)
		Manufactured stove	Wood	58 (77%)
China	Beijing	Electric stove	Electricity	8 (7%)
		Gas stove	Gas	101 (93%)
	Inner Mongolia	Manufactured stove	Coal	32 (26%)
		Electric stove	Electricity	38 (31%)

		Gas stove	Gas	24 (20%)
		Manufactured stove	Wood	22 (18%)
	Jiangsu	Manufactured stove	Ag/crop	14 (11%)
		Gas stove	Gas	78 (62%)
		Manufactured stove	Wood	34 (27%)
	Jiangxi	Gas stove	Gas	97 (98%)
	Liaoning	Manufactured stove	Ag/crop	80 (68%)
		Electric stove	Electricity	10 (9%)
		Gas stove	Gas	20 (17%)
		Manufactured stove	Shrub/grass	9 (8%)
	Qinghai	Manufactured stove	Coal	105 (98%)
	Shaanxi	Mud	Ag/crop	4 (3%)
		Manufactured stove	Ag/crop	14 (11%)
		Electric stove	Electricity	52 (40%)
		Gas stove	Gas	52 (40%)
	Shandong	Manufactured stove	Coal	16 (12%)
		Electric stove	Electricity	70 (55%)
		Gas stove	Gas	34 (27%)
		Manufactured stove	Wood	4 (3%)
	Shanxi	Mud	Coal	44 (56%)
		Manufactured stove	Coal	10 (13%)
		Electric stove	Electricity	22 (28%)
		Gas stove	Gas	3 (4%)
	Sichuan	Electric stove	Electricity	14 (13%)
		Gas stove	Gas	67 (63%)
		Manufactured stove	Wood	24 (23%)
	Yunnan	Electric stove	Electricity	18 (15%)
		Manufactured stove	Wood	103 (85%)
Colombia	Santander	Gas stove	Gas	30 (39%)
		Open fire	Wood	47 (61%)
India	Bangalore	Gas stove	Gas	14 (8%)
		Mud	Wood	185 (93%)
	Chandigarh	Mud	Animal dung	80 (57%)
		Gas stove	Gas	27 (19%)
		Mud	Wood	31 (22%)
	Chennai	Gas stove	Gas	141 (88%)
		Mud	Wood	17 (11%)
	Jaipur	Gas stove	Gas	71 (41%)
		Mud	Wood	94 (55%)
		Open fire	Wood	7 (4%)
	Trivandrum	Gas stove	Gas	89 (65%)
		Mud	Wood	20 (15%)
		Manufactured stove	Wood	28 (21%)
Pakistan	Karachi	Open fire	Wood	128 (97%)
Tanzania	Moshi	Open fire	Wood	17 (77%)
Zimbabwe	Harare	Open fire	Wood	51 (91%)

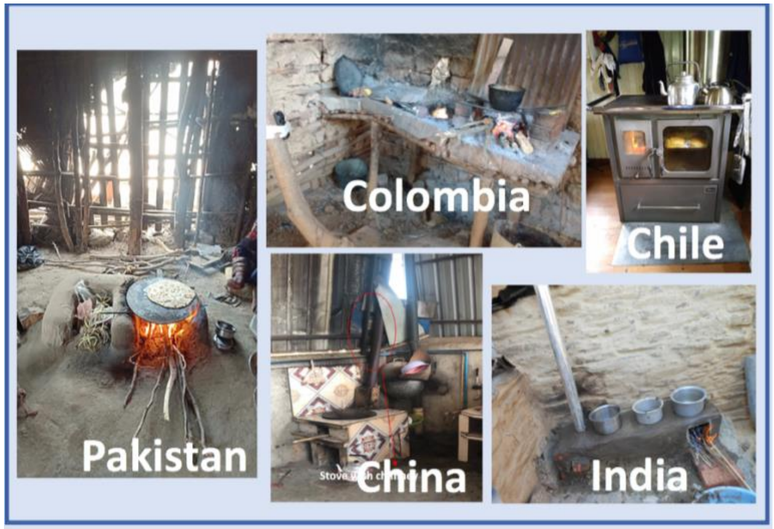

Figure S7. Common stove technologies for burning wood fuel commonly used among households in rural communities of PURE-AIR study countries.

Personal Exposures

Personal samples were collected among 998 individuals in a subset of ~25% of households (simultaneously with kitchen monitoring) (Table S4). Convenience sampling was used when selecting individuals personal sampling; field teams selected males and females from households selected for kitchen monitoring from each primary fuel group until the target sample was achieved for each sex in the community (priority was given to ‘paired’ measurements of males and females from the same households, when possible).

Table S4. Characteristics of individuals included in PURE-AIR study by primary cooking fuel.

	Primary Fuel Type															
	All Homes		Gas		Electric		Coal		Ag/crop residue		Wood		Animal dung		Shrub/ grass	
Sex	M	F	M	F	M	F	M	F	M	F	M	F	M	F	M	F
N	446	558	170	195	57	59	35	36	25	29	144	204	7	16	9	19
**Country/Region (N)**																
China	247 (55%)	264 (47%)	84 (49%)	89 (46%)	56 (98%)	56 (95%)	34 (97%)	36 (100%)	21 (84%)	23 (79%)	47 (33%)	55 (27%)	-	1 (6%)	3 (33%)	4 (21%)
India	125 (28%)	166 (30%)	69 (42%)	91 (47%)	-	-	-	1 (3%)	1 (4%)	1 (3%)	46 (32%)	59 (29%)	5 (71%)	11 (65%)	-	-
Other South Asia (Bangladesh, Pakistan)	28 (6%)	52 (9%)	-	-	-	-	-	-	3 (12%)	5 (18%)	17 (10%)	27 (11%)	2 (29%)	5 (29%)	6 (67%)	15 (71%)
South America (Chile, Colombia)	26 (6%)	38 (7%)	14 (8%)	14 (6%)	-	-	-	-	-	-	12 (8%)	23 (11%)	-	-	-	-
Africa (Tanzania, Zimbabwe)	20 (5%)	40 (7%)	1 (1%)	-	1 (2%)	3 (5%)	-	-	-	-	17 (12%)	30 (15%)	-	-	-	-
Age; Mean (SD)	61 (10)	59 (10)	61 (9)	59 (10)	62 (9)	59 (10)	62 (9)	57 (10)	62 (9)	60 (10)	61 (10)	59 (10)	62 (11)	60 (11)	63 (10)	57 (9)
Hours in kitchen; Mean (SD)	0·7 (0·9)	1·9 (1·5)	0·5 (0·6)	1·7 (1·4)	0·8 (0·7)	1·9 (1·0)	0·4 (0·6)	1·8 (1·0)	0·8 (0·9)	1·3 (0·8)	0·9 (1·3)	2·2 (1·6)	0·2 (0·2)	2·8 (1·9)	0·9 (1·0)	1·8 (0·9)
**Occupation [1] (%)**																
Homemaker	44 (11%)	262 (45%)	14 (5%)	86 (44%)	8 (14%)	14 (24%)	4 (12%)	12 (32%)	0	11 (38%)	18 (12%)	106 (51%)	0	16 (94%)	0	16 (84%)
Unskilled	259 (64%)	221 (43%)	98 (58%)	82 (42%)	41 (72%)	45 (76%)	27 (79%)	24 (73%)	20 (80%)	18 (62%)	64 (45%)	47 (23%)	1 (20%)	1 (6%)	4 (44%)	3 (16%)
Skilled	86 (21%)	19 (4%)	37 (22%)	6 (3%)	5 (8%)	0	2 (6%)	0	5 (20%)	0	28 (20%)	13 (6%)	5 (60%)	0	4 (44%)	0
Professional	18 (4%)	9 (2%)	9 (5%)	6 (3%)	2 (3%)	0	0	1 (3%)	0	0	3 (2%)	2 (1%)	1 (20%)	0	1 (11%)	0
Occupation air pollution exposure [2] (%)	138 (31%)	139 (25%)	49 (29%)	35 (18%)	7 (12%)	4 (7%)	2 (6%)	1 (3%)	1 (4%)	0	72 (51%)	90 (45%)	2 (29%)	5 (29%)	5 (55%)	4 (21%)
**Smoker (%)**	172 (39%)	13 (2%)	67 (40%)	4 (2%)	31 (54%)	1 (2%)	7 (21%)	1 (3%)	10 (40%)	1 (3%)	53 (37%)	6 (3%)	2 (29%)	-	2 (22%)	1 (5%)
2nd hand smoke exposure (%)	212 (48%)	195 (35%)	75 (45%)	55 (28%)	28 (49%)	26 (44%)	6 (18%)	9 (24%)	15 (60%)	11 (38%)	78 (55%)	77 (38%)	4 (57%)	5 (29%)	6 (67%)	12 (63%)

1. Occupation categories: (1) Professional= senior official/manager, professional, technician/associate professional, (2) Skilled worker= clerk, skilled agricultural or fishery worker, craft/related trade worker, (3) Unskilled worker= plant/machine operator, elementary occupation, (4) Homemaker. Percentages for occupation do not add up to 100% due to nonresponse (9%)
2. Occupational air pollution exposure represents participants that self-reported being exposed to specific air pollution sources (e.g. fires, industrial processes, traffic) at work during the 48-hour monitoring

A sensitivity analysis using chi-square tests of independence to compare cooking fuel use and socioeconomic characteristics of the subset of households selected for personal sampling (n=698) and the entire PURE-AIR household sample (n=2,541) showed no statistically significant differences in primary or secondary cooking fuel type used between the two samples (Table S5). The sample that received personal monitoring had slightly higher SES (p=0·01) than the full PURE-AIR sample, suggesting that personal exposures may be slightly underestimated compared to the full PURE-AIR sample. However, the aim of the PURE-AIR study was not to be representative at community or national levels, but to capture variation in the HAP exposure profiles among all types of cooking fuels used in PURE communities.

Table S5. Comparison of cooking fuel and socioeconomic characteristics among full PURE-AIR sample (n=2,451) and subset selected for personal monitoring (n=698)

	Full PURE-AIR sample (n=2,541)	PURE-AIR Households selected for personal monitoring (n=698)	X_2 test statistic (P-value)
**Primary Fuel Type**			
Gas	869 (34%)	259 (37%)	11·4 (0·12)
Electric	236 (9%)	74 (11%)	
Coal	209 (8%)	40 (6%)	
Charcoal	8 (1%)	2 (0%)	
Ag/crop residue	144 (6%)	35 (5%)	
Wood	903 (35%)	246 (35%)	
Animal dung	103 (4%)	18 (3%)	
Shrubs/grass	69 (3%)	24 (3%)	
Fuel stacking (%)	982 (39%)	370 (37%)	0·73 (0·39)
**Secondary fuel (%)**			
None	1556 (61%)	446 (62%)	7·1 (0·62)
Gas	409 (16%)	101 (14%)	
Electric	314 (12%)	86 (12%)	
Coal	17 (1%)	6 (1%)	
Charcoal	5 (0%)	3 (0%)	
Ag/crop residue	23 (1%)	6 (1%)	
Wood	198 (8%)	59 (8%)	
Animal dung	14 (1%)	8 (1%)	
Shrubs/grass	4 (0%)	2 (0%)	
Outdoor cooking (%)	418 (17%)	109 (16%)	0·31 (0·57)
**Heating type (%)**			
No heating	1,695 (67%)	437 (63%)	56·7 (<0·001)*
Electric/gas	195 (8%)	81 (11%)	
Mud stove	263 (10%)	30 (4%)	
Open fire	299 (12%)	132 (19%)	
Chimney stove	82 (3%)	15 (2%)	
Smoking in home (%)	708 (28%)	221 (32%)	3·83 (0·05)
**Home Asset Index[1] (%)**			
Tertile 1 (Lowest)	1,323 (70%)	333 (48%)	9·4 (0·01)
Tertile 2	819 (26%)	274 (39%)	
Tertile 3 (Highest)	318 (13%)	91 (13%)	
**Education Level[2] (%)**			
None	575 (24%)	130 (19%)	11·3 (0·01)
Primary	802 (34%)	267 (38%)	
Secondary	962 (40%)	281 (40%)	
Trade/university	79 (3%)	18 (3%)	

*Note: Education level not available for all households; numbers do not add up to n=2,541

At the discretion of each participant, the UPAS could be worn in a harness or armband (Figure S8); the majority (~80%) of participants selected to wear the UPAS in an armband (likely because it was less invasive than a harness and prevented their clothing from sticking to them in high temperatures (which could be an issue with the harness). We conducted a sensitivity analysis to compare exposures between both UPAS wearing locations in PURE-AIR sub-national regions where at least 5 male or female participants using the same primary cooking fuel during exposure monitoring elected to wear the harness.

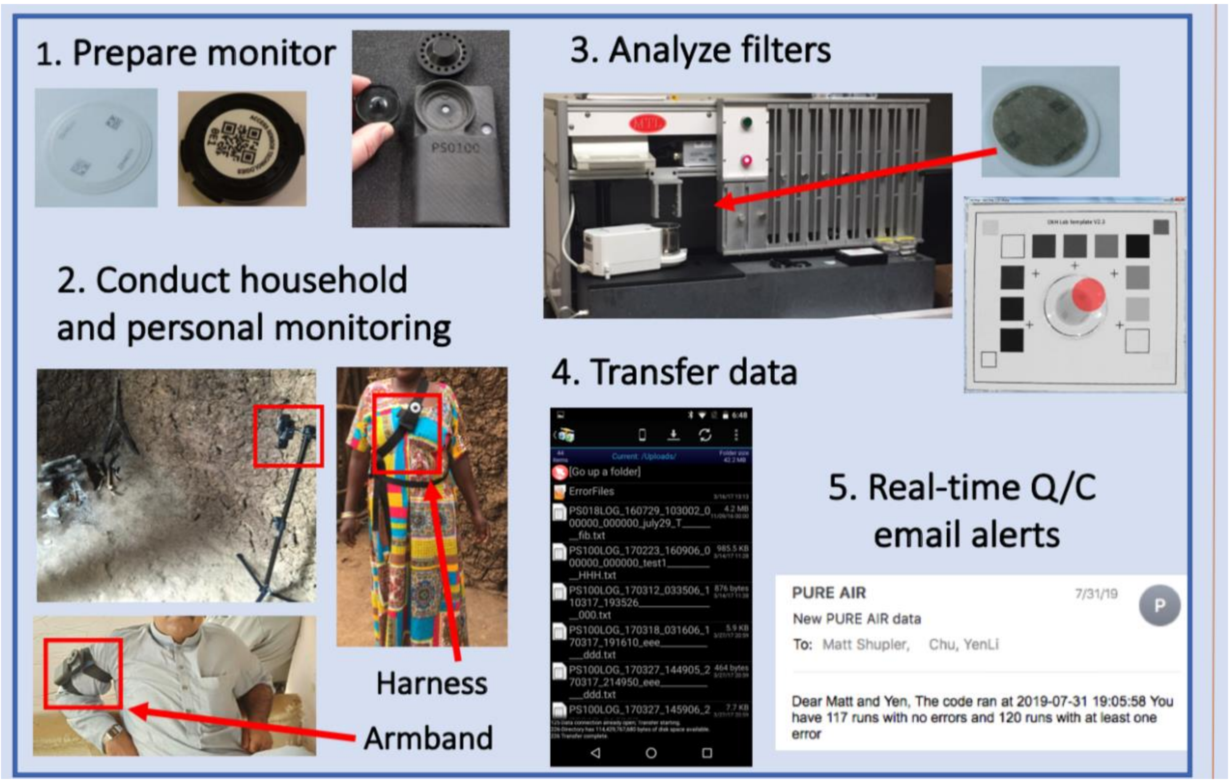

Figure S8. Work flow for PURE-AIR study.

There were no significant differences in average sex- and fuel-specific 48 hour exposures in the six sub-national regions examined (Table S6). The exception was among males cooking with agricultural/crop waste in Liaoning, China. We hypothesize that this may have been a chance event due to no significant differences between corresponding female exposures in Liaoning. While the point estimate of exposure may vary by ~20 µg/m^3 depending on the where the UPAS was worn, the direction of the difference (e.g. harness having higher or lower average values than the armband) varied between locations and between sexes within the same location, suggesting a lower risk of differential exposure misclassification.

Table S6. Average 48-hour $PM_{2.5}$ exposures by UPAS wearing method.

**Country**	**Sub-National Region**	**Primary Cooking Fuel**	**Sex**	**Wearing Method**	**N**	**Average 48-hour $PM_{2.5}$ Exposure (95% CI) (µg/m^3)**
China	Beijing	Gas	Female	Harness	9	75 (34,163)
				Armband	8	52 (31,86)
			Male	Harness	5	45 (21,97)
				Armband	10	73 (41,131)
	Liaoning	Agricultural/ crop waste	Female	Harness	9	69 (47,101)
				Armband	5	90 (46,173)
			Male	Harness	7	140 (102,194)

				Armband	7	42 (28,63)
	Qinghai	Coal	Female	Harness	15	65 (44,96)
				Armband	6	97 (59,162)
India	Chandigarh	Gas	Female	Harness	5	153 (138,170)
				Armband	7	154 (111,213)
	Chennai	Gas	Female	Harness	23	61 (50,74)
				Armband	27	42 (31,55)
			Male	Harness	15	63 (50,80)
				Armband	22	42 (32,55)
Zimbabwe	Harare	Wood	Female	Harness	9	198 (140,278)
				Armband	19	172 (127,233)

Filter Weighing Procedure

Filters (MTL PT37DMCAN-PF03, Measurement Technology Laboratories, Bloomington, Minnesota) were weighed using the MTL Filter Weighing System (Figure S8), which uses a robotic arm to carry filters from storage silos to the balance. The balance used was an MT5 analytical microbalance (Mettler-Toledo, Columbus, Ohio), capable of weighing to 1 microgram. The balance was set atop a granite block for added stability. Quality control was tracked by weighing 3 calibration weights (100mg, 200mg, and 400mg) in addition to 5 reference filters prior to weighing any sample filters. Environmental conditions were stabilized within the following ranges: 30-40% relative humidity, 95-105 kPa, and 21-25 C with target values of 35.00% humidity, 100 kPa, and 23 C, respectively. The temperature and relative humidity conditions were controlled by an environmental control unit by m.a.n systems (model hcdh-01). A Vaisala Humidity and Temperature Transmitter HMT333 and Vaisala PTB330 Digital Barometer are built into the weighing system, so that if environmental conditions deviate from the above values, weighing is ceased until environmental tolerance is achieved for at least 60 minutes.

Filter samples were given at least 48 hours to acclimatize to the ambient environmental conditions before weighing, both after unpackaging and upon re-arrival to the lab. Each filter weight was performed in triplicate, with a criteria that the standard deviation must be less than 2.00 ug. Duplicate weights were also performed as a quality control measure, occurring for the first filter, the last filter, and every 20th filter weighed.

Each filter was printed with a unique 9-digit numerical ID and a corresponding QR code. The filter weighing system has a built-in barcode reader that scans this QR code prior to weighing, and assigns a weight to each filter based off this unique ID. For added redundancy, each filter is placed in a metal carrier with a unique 7-digit alphanumeric ID prior to loading it in the filter weighing system. If the scanner is unable to scan the filter ID, it scans the carrier ID and assigns the filter weight to that carrier, which can be then assigned to the corresponding filter ID post-weighing. Prior to leaving for sampling, each filter was placed in a cassette with 3 unique alphanumeric characters. The filter ID and cassette IDs were logged in both the filter weighing system database and an Excel spreadsheet for added redundancy. When the filter was returned post-sampling, the cassette containing the filter was opened and checked against our records ensure that the filter had not been switched in the field. Visual observations for changes in the filter that might affect the integrity of the data, such as holes, were recorded as well.
A report was generated in Excel with the following information for each filter: the filter ID, the date and time the filter was weighted pre- and post-sampling, each triplicate weight value pre- and post-sampling, the standard deviation between the three triplicate pre- and post-sampling weights, the average pre- and post-sampling weight, net weight, the cassette ID the filter left and returned to the lab in, and any comments on the condition of the filter.

Relationship between $PM_{2.5}$ Personal Exposures and Kitchen Concentrations

The relationship between $PM_{2.5}$ personal exposures and kitchen concentrations was modeled on a log scale (linear regression equations provided were back-transformed to units of $\mu g/m^3$). Exposures and concentrations were moderately correlated (Spearman correlation r=0.69) (Figure S9a), however the strength of association varied by country. Exposures and concentrations were most strongly correlated in Chile and India (r>0·7) and less in Zimbabwe and Bangladesh (r<0·3) (Figure S9b).

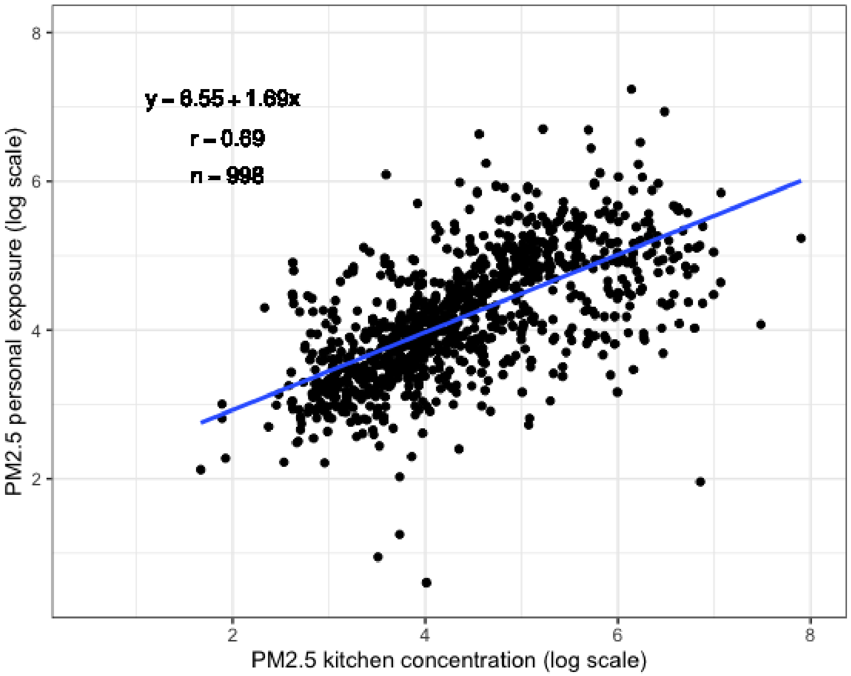

Figure S9a. Relationship between $PM_{2.5}$ kitchen concentrations and personal exposures in rural PURE-AIR communities. Note: Regression equations are reported in units of $\mu g/m^3$.

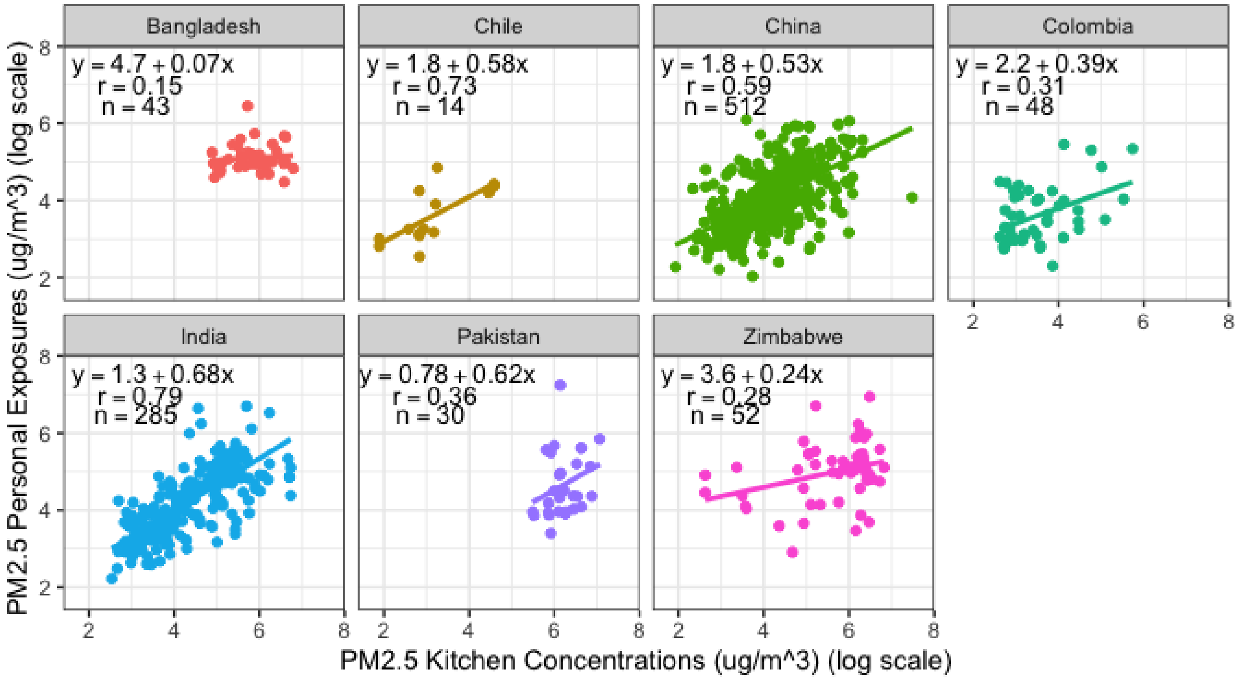

Figure S9b. Relationship between $PM_{2.5}$ kitchen concentrations (log scale) and personal exposures (log scale) in rural PURE-AIR communities by country. Note: Regression equations are reported in units of $\mu g/m^3$. Tanzania not included due to low sample size.

Heating Fuels

In six sub-national regions across four countries where the type of heating method varied among households using the same primary cooking fuel type, the effect of household heating on average concentrations was examined. The analysis revealed that heating with polluting fuels in mud stoves or open fires significantly increased average kitchen concentrations among households cooking with gas as their primary cooking fuel (Figure S10). No significant difference in average $PM_{2.5}$ concentrations was found by use of polluting heating fuels among households cooking with wood.

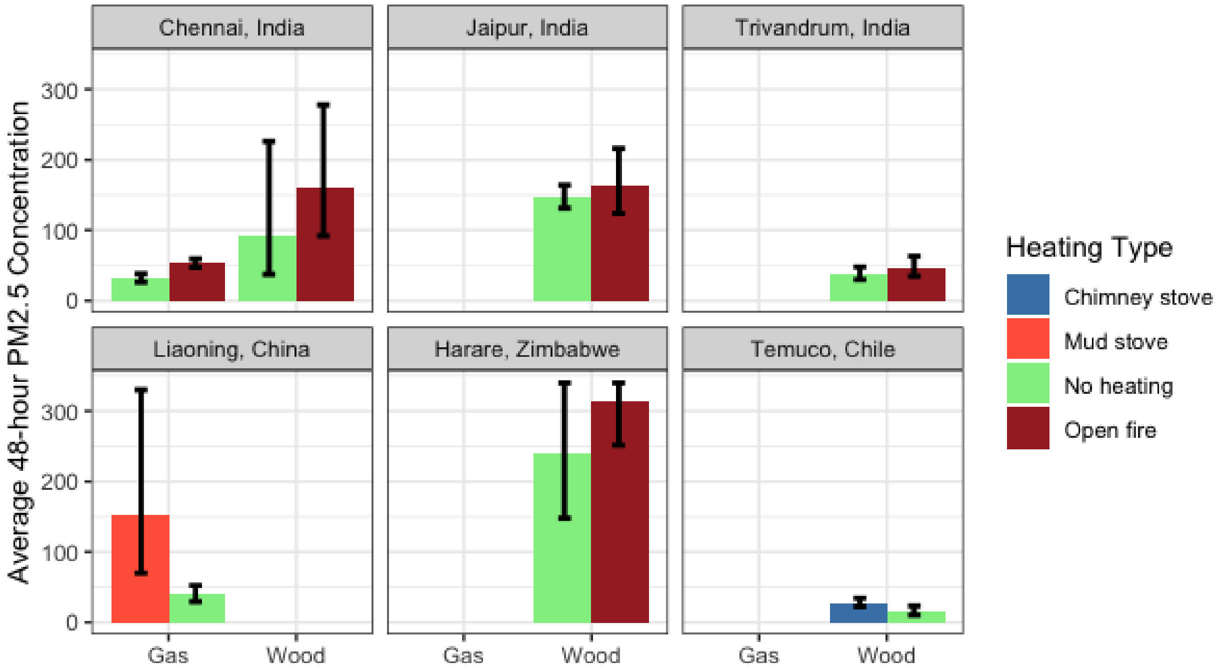

Figure S10. Average 48-hour $PM_{2.5}$ concentration in rural PURE-AIR communities by stove type used for heating and primary cooking fuel (in six sub-national regions with variations in heating type).

Exposure Ratios

Many measurement campaigns, epidemiologic studies and assessment tools focus on kitchen concentrations that are logistically easier to collect, especially for large populations and over long time periods. However, exposure measurements are more relevant from a health perspective. We sought to assess these relationships to enable others to estimate exposures from kitchen measurements as has been done in several impact assessment analyses (e.g. HAPIT, Global Burden of Disease). Male:female and personal:kitchen ratios from 227 households with paired male/female samples (N=454 measurements) were compared to exposure ratios from all 998 personal measurements. The exposure ratios were similar between each sample (Table S7).

Table S7. Comparison of exposure ratios among entire PURE-AIR personal sample (n=998) ('All') and only samples with male-female pairs collected from the same PURE household (n=454) ('Paired Only') .

Continent	Country	Fuel Type	All Female: Kitchen	Paired Only Female: Kitchen	All Male: Kitchen	Paired Only Male: Kitchen
All	All	All Fuels	0.89	0.89	0.92	0.89
		Gas	1.07	0.96	1.02	0.94
		Electric	1.22	1.01	1.08	0.98
		Coal	0.98	1.06	0.99	1.03
		Ag/crop	0.83	1.03	0.94	0.94
		Wood	0.71	0.71	0.68	0.68

		Animal dung	0.77	0.98	0.88	1.05
		Shrub/grass	0.50	0.43	0.46	0.50
South America	Chile	Wood	1.31	1.33	2.94	1.68
	Colombia	Gas	1.05	1.13	1.65	3.68
		Wood	0.57	1.07	0.50	0.57
Asia	Bangladesh	Ag/crop	0.58	0.57	0.62	0.63
		Shrub/grass	0.50	0.43	0.38	0.27
		Wood	0.61	0.50	0.56	0.43
	China	Ag/crop	0.97	1.11	0.8	1.16
		Coal	0.98	1.06	1.02	1.03
		Electric	1.22	1.01	1.11	0.98
		Gas	1.01	0.80	1.09	0.81
		Wood	0.89	0.81	0.9	0.72
	India	Animal dung	0.77	1.10	0.74	1.20
		Gas	1.14	1.01	0.94	1.01
		Wood	0.88	0.71	0.85	0.94
	Pakistan	Wood	0.32	0.33	0.22	0.15
Africa	Zimbabwe	Wood	0.56	0.43	0.52	0.22

Slightly higher $PM_{2.5}$ personal:kitchen exposure ratios were observed compared to BC exposure ratios across all polluting primary fuels (Table S8). This suggests that sources other than biomass combustion, which do not emit BC, likely contributed to $PM_{2.5}$ exposures. It is concluded that the observed $PM_{2.5}$ exposures in PURE-AIR are not entirely due to HAP.

Country-specific $PM_{2.5}$ and BC kichen:personal exposure ratios varied substantially. Median female:kitchen exposure ratios ranged from 0·1-1·1 among wood primary fuel-using households across countries. Median country-level $PM_{2.5}$ male:female exposure ratios also had a large range: generally ≥1·0 in India, China and South American countries, while ≤0·7 in Pakistan, Bangladesh, and African countries.

Table S8. Exposure ratios among paired samples (N=454; 227 male/female pairs) by country and primary fuel type.

			$PM_{2.5}$									BC								
**Country**	**Fuel Type**	**N**	**Female : Kitchen**			**Male : Kitchen**			**Male : Female**			**Female : Kitchen**			**Male : Kitchen**			**Male : Female**		
			**Med[1]**	**GM[2]**	**95% CI**	**Med[1]**	**GM[2]**	**95% CI**	**Med[1]**	**GM[2]**	**95% CI**	**Med[1]**	**GM[2]**	**95% CI**	**Med[1]**	**GM[2]**	**95% CI**	**Med[1]**	**GM[2]**	**95% CI**
All	All Fuels	227	0·89	0·82	(0·74,0·91)	0·89	0·79	(0·71,0·88)	1·00	0·97	(0·89,1·06)	0·86	0·68	(0·46,1·02)	0·86	0·64	(0·45,0·92)	1·00	0·94	(0·67,1·32)
	Ag/crop	15	1·03	1·1	(0·96,1·25)	0·94	1·27	(1·14,1·42)	1·06	1·16	(1·06,1·26)	0·92	1·13	(0·97,1·33)	0·95	1·41	(1·2,1·66)	1·09	1·24	(1·15,1·35)
	Animal dung	2	0·98	1·09	(1·07,1·11)	1·05	1·19	(1·16,1·21)	1·07	1·09	(1·09,1·09)	0·87	0·87	(0·87,0·88)	0·98	0·98	(0·97,0·99)	1·12	1·12	(1·11,1·12)
	Coal	23	1·06	1·05	(0·94,1·18)	1·03	0·86	(0·79,0·93)	0·94	0·82	(0·75,0·88)	0·84	1·82	(1·13,2·93)	0·89	1·59	(0·97,2·6)	0·95	0·87	(0·8,0·95)
	Electric	25	0·80	0·80	(0·73,0·87)	0·81	0·82	(0·75,0·89)	1·02	1·03	(0·95,1·11)	0·85	0·37	(0·24,0·58)	0·92	0·89	(0·82,0·97)	1·20	2·41	(1·54,3·75)
	Gas	85	0·96	0·91	(0·85,0·98)	0·94	0·96	(0·89,1·05)	1·03	1·06	(0·99,1·13)	0·98	0·99	(0·92,1·05)	0·98	0·98	(0·91,1·05)	1·04	0·99	(0·91,1·08)
	Shrub/grass	5	0·43	0·60	(0·56,0·65)	0·50	0·46	(0·4,0·52)	0·87	0·76	(0·7,0·82)	0·60	0·65	(0·6,0·7)	0·39	0·43	(0·38,0·49)	0·75	0·66	(0·62,0·71)
	Wood	79	0·71	0·67	(0·6,0·75)	0·68	0·56	(0·49,0·64)	0·93	0·84	(0·75,0·94)	0·56	0·34	(0·24,0·5)	0·48	0·25	(0·17,0·36)	0·91	0·73	(0·65,0·82)
Chile	Wood	4	1·33	1·44	(1·34,1·55)	1·68	1·60	(1·44,1·78)	0·95	1·11	(1·01,1·22)	0·92	1·85	(1·52,2·26)	1·03	1·53	(1·32,1·77)	0·88	0·82	(0·77,0·88)
Colombia	All Fuels	6	1·13	1·05	(0·99,1·11)	1·40	1·61	(1·4,1·86)	1·35	1·53	(1·35,1·73)	0·82	0·94	(0·87,1·01)	0·65	0·66	(0·59,0·74)	0·85	0·71	(0·65,0·76)
	Gas	4	1·13	1·14	(1·11,1·17)	3·68	2·74	(2·42,3·1)	2·97	2·4	(2·18,2·65)	0·98	1·07	(0·98,1·17)	0·77	0·99	(0·9,1·08)	1·05	0·92	(0·88,0·97)
	Wood	2	1·07	0·89	(0·8,1)	0·57	0·56	(0·54,0·58)	0·88	0·62	(0·58,0·67)	0·73	0·72	(0·7,0·74)	0·31	0·3	(0·28,0·32)	0·46	0·41	(0·38,0·45)
Bangladesh	All Fuels	7	0·44	0·53	(0·51,0·55)	0·39	0·38	(0·35,0·42)	0·87	0·72	(0·68,0·77)	0·39	0·41	(0·38,0·43)	0·36	0·3	(0·27,0·32)	0·89	0·73	(0·69,0·77)
	Ag/crop	2	0·57	0·56	(0·54,0·58)	0·63	0·58	(0·54,0·62)	1·05	1·04	(1·01,1·07)	0·48	0·47	(0·45,0·49)	0·44	0·43	(0·42,0·44)	0·92	0·91	(0·91,0·92)
	Shrub/grass	3	0·43	0·54	(0·51,0·57)	0·27	0·3	(0·26,0·34)	0·63	0·55	(0·51,0·59)	0·45	0·49	(0·46,0·52)	0·25	0·28	(0·25,0·31)	0·75	0·58	(0·53,0·62)
	Wood	2	0·50	0·48	(0·46,0·5)	0·43	0·36	(0·32,0·4)	0·8	0·75	(0·7,0·8)	0·29	0·27	(0·25,0·29)	0·27	0·22	(0·2,0·25)	0·86	0·83	(0·8,0·87)
China	All Fuels	132	0·88	0·84	(0·75,0·93)	0·91	0·87	(0·79,0·94)	1·00	1·03	(0·95,1·12)	0·92	0·88	(0·65,1·18)	0·92	1·00	(0·8,1·25)	1·04	1·14	(0·92,1·41)
	Ag/crop	13	1·11	1·22	(1·06,1·39)	1·16	1·43	(1·28,1·6)	1·07	1·18	(1·07,1·29)	1·20	1·30	(1·1,1·53)	1·08	1·69	(1·44,1·99)	1·19	1·30	(1·2,1·42)
	Coal	23	1·06	1·05	(0·94,1·18)	1·03	0·86	(0·79,0·93)	0·94	0·82	(0·75,0·88)	0·84	1·82	(1·13,2·93)	0·89	1·59	(0·97,2·6)	0·95	0·87	(0·8,0·95)
	Electric	25	0·80	0·8	(0·73,0·87)	0·81	0·82	(0·75,0·89)	1·02	1·03	(0·95,1·11)	0·85	0·37	(0·24,0·58)	0·92	0·89	(0·82,0·97)	1·20	2·41	(1·54,3·75)
	Gas	44	0·85	0·71	(0·64,0·8)	1·01	0·88	(0·79,0·97)	1·23	1·23	(1·22,1·24)	0·99	0·99	(0·91,1·06)	0·93	0·92	(0·85,1)	0·98	0·93	(0·85,1·03)
	Shrub/grass	2	0·81	0·72	(0·67,0·78)	0·72	0·69	(0·65,0·73)	1·01	0·96	(0·9,1·03)	1·11	0·99	(0·91,1·08)	1·04	0·81	(0·71,0·93)	0·84	0·82	(0·79,0·86)
	Wood	25	1·11	1·22	(1·06,1·39)	1·16	1·43	(1·28,1·6)	1·07	1·18	(1·07,1·29)	0·86	0·69	(0·62,0·76)	0·68	0·64	(0·59,0·69)	1·00	0·94	(0·87,1·01)
India	All Fuels	67	0·96	0·93	(0·86,1)	1·02	0·96	(0·88,1·04)	1·06	1·04	(0·96,1·12)	0·88	0·92	(0·87,0·98)	0·95	1·04	(0·98,1·11)	1·10	1·13	(1·06,1·2)
	Animal dung	2	1·1	1·09	(1·07,1·11)	1·20	1·19	(1·16,1·21)	1·09	1·09	(1·09,1·09)	0·87	0·87	(0·87,0·88)	0·98	0·98	(0·97,0·99)	1·12	1·12	(1·11,1·12)
	Gas	37	1·01	1·04	(0·99,1·09)	1·01	1·04	(0·98,1·1)	1·04	1·00	(0·96,1·04)	0·96	0·97	(0·93,1·02)	1·00	1·10	(1·04,1·15)	1·11	1·13	(1·05,1·21)
	Wood	28	0·71	0·78	(0·71,0·87)	0·94	0·85	(0·76,0·95)	1·09	1·08	(0·98,1·2)	0·52	0·62	(0·54,0·71)	0·54	0·71	(0·63,0·81)	1·00	1·15	(1·13,1·18)
Pakistan	Wood	8	0·33	0·37	(0·32,0·43)	0·15	0·14	(0·13,0·14)	0·43	0·36	(0·32,0·42)	0·18	0·16	(0·14,0·18)	0·05	0·05	(0·05,0·06)	0·24	0·34	(0·3,0·38)
Zimbabwe	Wood	9	0·43	0·47	(0·41,0·55)	0·22	0·26	(0·23,0·3)	0·71	0·55	(0·47,0·65)	0·38	0·04	(0·02,0·1)	0·19	0·03	(0·01,0·06)	0·98	0·66	(0·54,0·81)

1. Med = Median
2. GM = Geometric mean

Seasonality

A sensitivity analysis was conducted by dichotomizing average 48-hour primary fuel-specific $PM_{2.5}$ and BC concentrations according to wet (summer) and dry (winter) seasons. Wet season was assumed to fall between the months of April-September (inclusive) and dry season included October-March; the seasons were reversed for countries in the Southern Hemisphere (Chile, Tanzania, Zimbabwe). This method for assessing seasonality was used by the authors in a previous global HAP modeling paper.[2] One-third (8 out of 22) of sub-national regions were found to have had measurements that sufficiently spanned both seasons (<85% of measurements in one season; see bolded rows bolded in Table S9 for regions that met the criterion). Note: Shandong, China is not included as measurements only spanned two consecutive months.

Table S9. Number of household measurements in wet/dry season by PURE sub-national region.

Country	Sub-national region	Months of data collection	Total number of HH samples	Number (%) of HH samples in winter (dry) season (Oct-March)
China	Beijing	November	109	98 (90%)
China	Inner Mongolia	November, December	122	122 (100%)
China	Jiangsu	October, November	126	126 (100%)
China	Jiangxi	June, July	99	0
**China**	**Liaoning**	**May, December**	**119**	**99 (83%)**
China	Qinghai	August, September	107	0
China	Shaanxi	January, February, March	129	129 (100%)
China	Shandong	September, October	126	60 (48%)
China	Shanxi	May, July	79	0
China	Sichuan	March	106	106 (100%)
China	Yunnan	January	122	122 (100%)
India	Bangalore	July, August, September, October	199	30 (15%)
India	Chandigarh	November, December, January, February	140	129 (92%)
**India**	**Chennai**	**July, August, September, October, December**	**161**	**74 (46%)**
**India**	**Jaipur**	**November, December, January, February, March, April, May**	**172**	**135 (78%)**
India	Trivandrum	April, May, June, July, August, September	137	0
**Chile**	**Temuco**	**March, June, July, August, October, November**	**75**	**32 (43%)**
**Colombia**	**Santander**	**December, January, February, April**	**77**	**53 (69%)**
Bangladesh	Dhaka	November, December, January	126	126 (100%)
**Pakistan**	**Karachi**	**January, February, March, April, July, September, October, November, December**	**132**	**91 (69%)**
**Tanzania**	**Moshi**	**March, May, June**	**22**	**6 (38%)**
**Zimbabwe**	**Harare**	**August, September, October, December, January, February, March, April**	**56**	**16 (29%)**

When stratifying the measurements by season in the eight sub-national regions, substantial differences in average $PM_{2.5}$ kitchen concentrations existed among households cooking with the same primary fuels in several locations (e.g. Jaipur, India; Karachi, Pakistan, Temuco, Chile) where measurements spanned winter and summer (Figure S11); minimal seasonal differences were observed in Santander, Colombia and Harare, Zimbabwe.

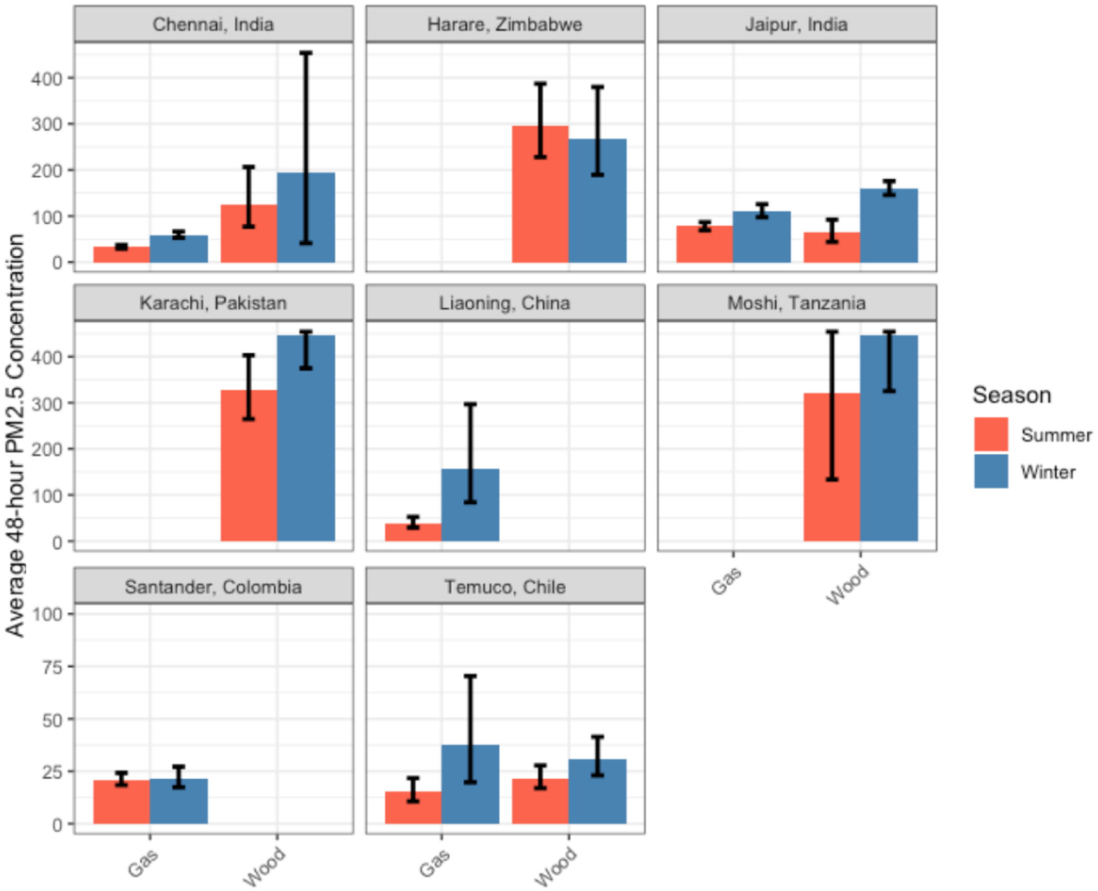

Figure S11. Summer (wet) and winter (dry) season average 48-hour household concentrations in rural PURE-AIR communities in sub-national regions where household measurements spanned both seasons.

In 24 households from two sub-national regions in China (Beijing, Liaoning) and India (Chennai, Jaipur), 48-hour monitoring was conducted approximately 6 months apart in winter months (November-February) and summer months (May-August) to assess seasonal concentration differences. Average 48-hour $PM_{2.5}$ kitchen concentrations were an average of 60 μg/m3 (55%) higher in winter than summer. The seasonal difference varied between sub-national regions: Chennai: 17 μg/m3, Jaipur: 112 μg/m3, Beijing: 68 μg/m3, Liaoning: 65 μg/m3. Among 8 of the 25 households where there was no change in heating fuel usage during the winter/summer months, average winter kitchen concentrations (144 μg/m3) remained consistently higher than average summer concentrations (67 μg/m3).

Kitchen Location

'Kitchen type' is a derived variable that was coded to match groupings reported in the WHO Harmonized survey for monitoring household energy use (https://www.who.int/airpollution/household/1_Harmonized_household_energy_survey_questions-list_format_final_Nov2019.pdf?ua=1). Participants that reported cooking indoors and having at least two rooms in the home were categorized as cooking indoors 'in a separate room'. Those reporting having one room in the home were categorized as indoor cooking with 'no separate room'. Participants that reported cooking inside with their kitchen being 'partially open to the outside' were categorized as cooking on a 'porch or veranda'. Those that reported cooking outdoors were assumed to cook 'in open air'.

The correlation between exposures and concentrations monotonically increased with decreasing ventilation in the kitchen (e.g. between $PM_{2.5}$ kitchen concentrations and female exposures: Spearman correlation (r) = 0·46 in

outdoor kitchens, r=0·66 in multi-room, indoor kitchens and r=0·80 in single-room, indoor kitchens (Figure S12). A similar relationship existed for BC absorbance (Figure S13).

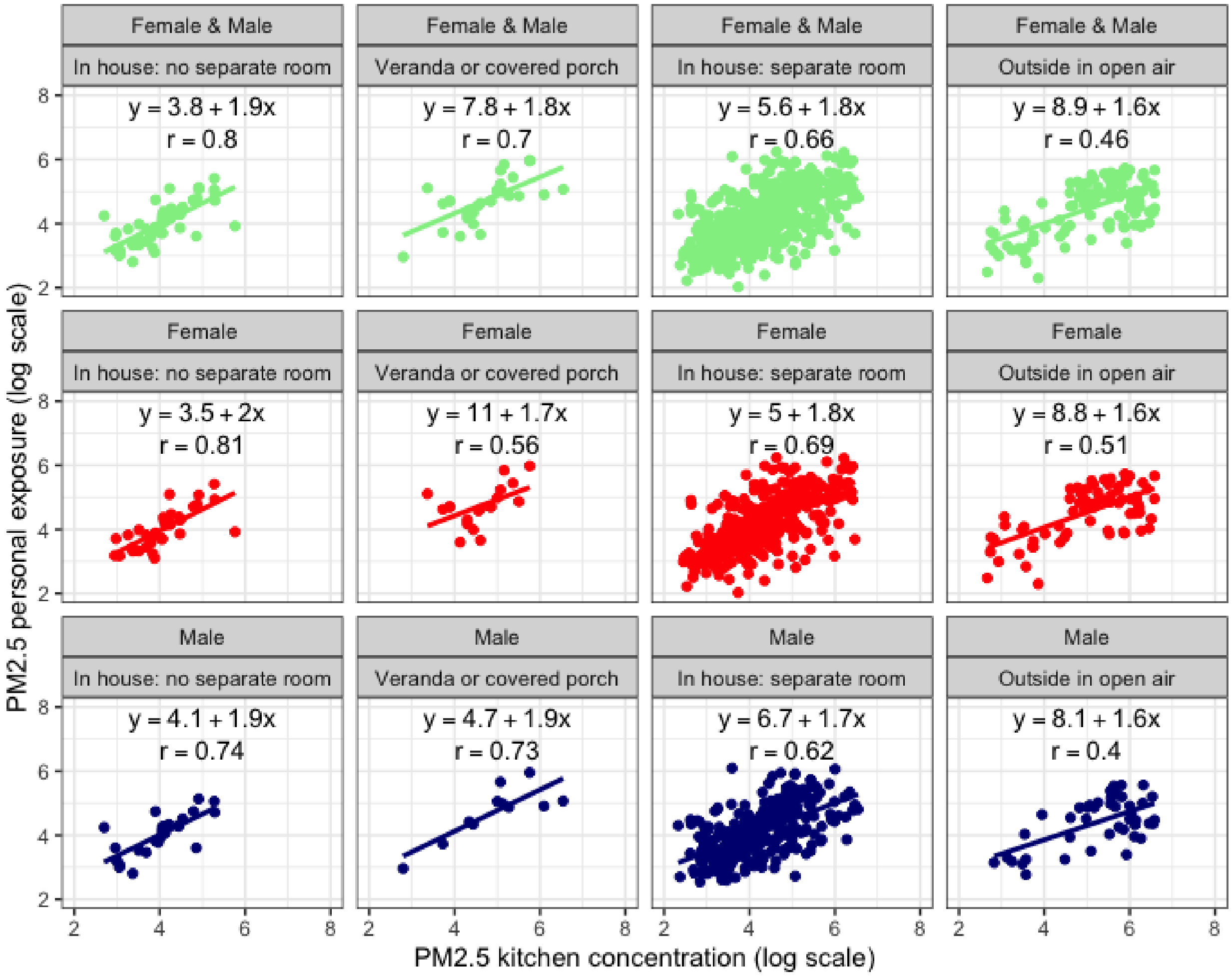

Figure S12. Scatterplot of average 48-hour $PM_{2.5}$ kitchen versus personal levels (log scale) in rural PURE-AIR communities by gender and kitchen type. Note: Regression equations are reported in units of μg/m^3. r reported in the figure is the Spearman correlation.

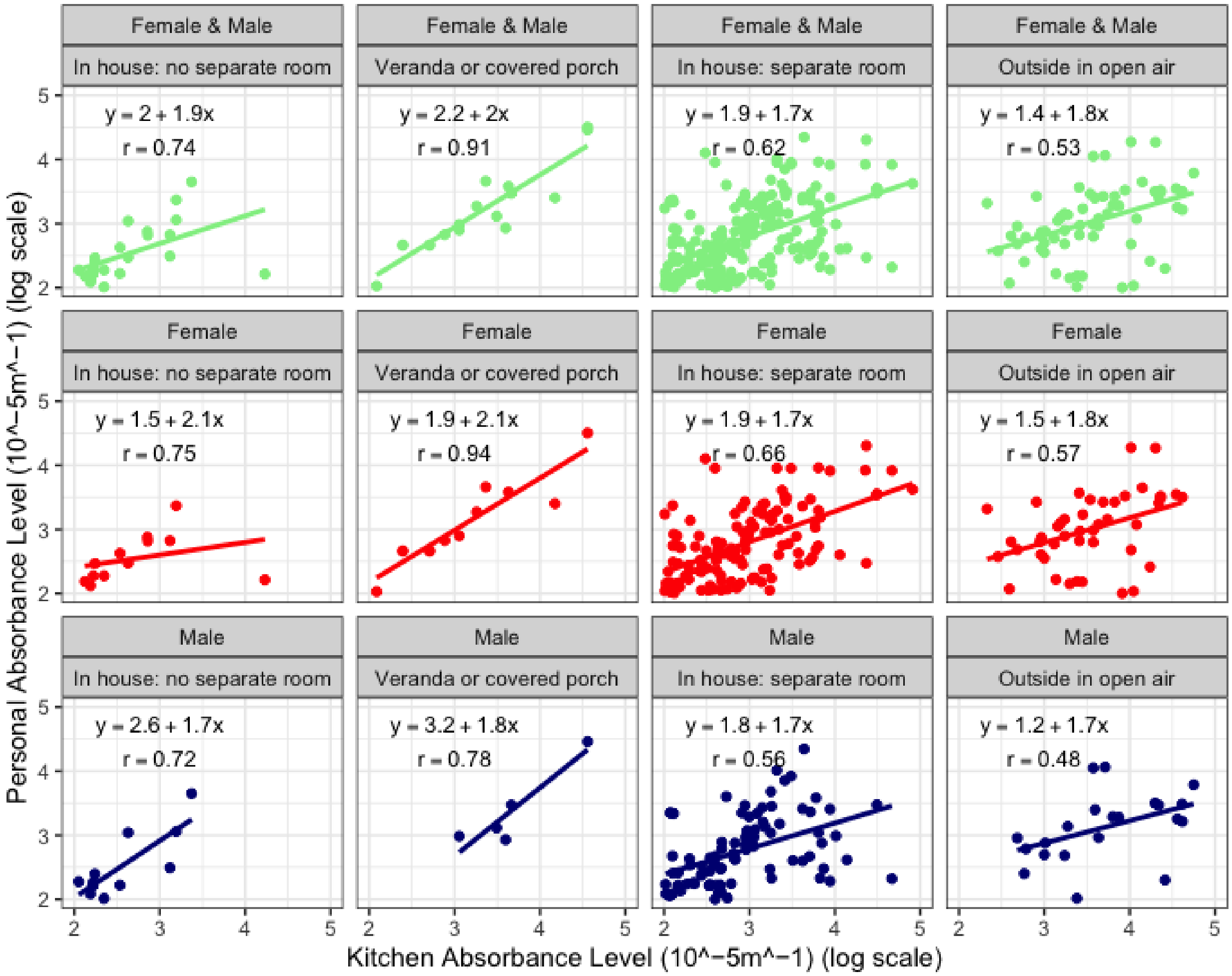

Figure S13. Scatterplot of average 48-hour BC absorbance kitchen versus personal measurements (log scale) in rural PURE-AIR communities by gender and kitchen type. Note: Regression equations are reported in units of $10^{-5}m^{-1}$.

UPAS Evaluation

To assessment sensitivity of UPAS measurements, two 48-hour kitchen samples were collected in duplicate in 25 total households in three countries (Pakistan, India, China). The duplicate samples showed high agreement (Spearman r=0·8) (Figure S14), with a median $PM_{2.5}$ concentration difference of 8·5 $\mu g/m^3$ (percent difference: 12.5%).

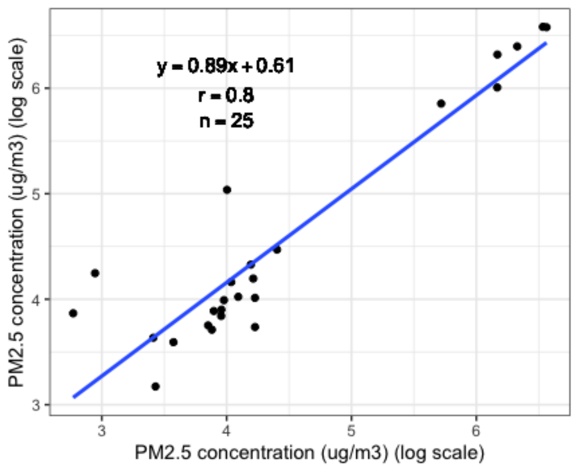

Figure S14. Relationship between $PM_{2.5}$ kitchen concentrations collected in duplicate from the UPAS (n=25). Note: Regression equation is reported in units of $\mu g/m^3$.

Black Carbon

Average absorbance levels varied greatly between countries among the same primary fuel type. For example, the average light absorbance among gas fuels in China ($2\cdot1\ 10^{-5}m^{-1}$) and India ($2\cdot7\ 10^{-5}m^{-1}$) was twice as high as among gas fuels in South American countries ($1\cdot1\ 10^{-5}m^{-1}$). In turn, kitchens in which primary polluting fuels were used had significantly higher BC concentrations in Bangladesh, Pakistan and African countries compared with China and India (Table S10). This country-level trend generally remained among female BC exposures, but deviated among male exposures, with average male BC levels being much lower in Pakistan and Bangladesh than in India among households using animal dung, signaling high ambient BC exposures in Chandigarh, India, where animal dung was predominantly used for cooking (Table S11).

In the two countries (China, Bangladesh) where the most polluting fuel (shrub/grass) was used for cooking, the average BC exposure of males in China was triple that of males in Bangladesh. However, BC exposures among Bangladeshi women cooking with shrub/grass was quintuple that of Chinese women (Table S11). A similar trend existed between personal BC exposures in Indian and Bangladeshi households cooking with animal dung.

Table S10. Summary of **average 48-hour BC absorbance levels ($10^{-5}m^{-1}$)** among kitchen samples by primary fuel type.

	Primary Fuel Type							
**Region**	**Gas**	**Electric**	**Coal**	**Charcoal**	**Ag/crop residue**	**Wood**	**Animal dung**	**Shrubs/ grass**
China	2·1 (2·0,2·3)	2·6 (2·3,2·9)	2·4 (2·1,2·7)	3·5 (1·5,8·3)	4·8 (4,5·7)	3·1 (2·8,3·5)	2·4 (0·3,17·5)	4·3 (2·6,7·1)
India	2·7 (2·5,3.0)				10·7 (3·8,30·3)	6·6 (5·9,7·4)	8·7 (7·8,9·8)	
Other South Asia[1]					25·5 (21·7,30·0)	25·0 (21·6,28·8)	25·6 (22·1,29·7)	38·3 (32·4,45·2)
South America[2]	1·1 (0·9,1·3)					2·1 (1·7,2·6)		
Africa[3]		0·5 (0·1,2·7)		1·4 (0·7,2·8)		13·3 (11·1,15·8)		
**Secondary fuel**								
None	2·1 (1·9,2·3)	2·5 (2·2,2·9)	2·5 (2·1,2·9)	2·8 (1·3,6·4)	9·4 (7·0,12·5)	8·3 (7·5,9·3)	18·5 (13·3,25·7)	33·5 (26·8,41·9)
Gas		3·9 (3·2,4·8)			4·1 (3·0,5·6)	4·3 (3·8,4·9)	8·5 (7·6,9·5)	18 (10·7,30·3)
Electric	2·3 (2·0,2·5)	3·1 (1·2,8·5)	1·8 (1·4,2·2)		4·9 (3·7,6·4)	3·3 (2·7,3·9)		4·6 (2·5,8·5)
Coal	6·0 (2·1,17·5)	2·0 (1·0,4·2)			12 (5·2,27·7)	6·2 (0·5,71·1)		
Charcoal	22·8 (6·8,77·0)							
Ag/crop waste	2·2 (1·6,3·0)	5·3 (3·6,7·7)				21·1 (17·3,25·7)		
Wood	2·6 (2·3,3·0)	1·4 (0·9,2·3)				18·2 (10·0,33·0)		
Animal dung	6·5 (4·5,9·3)				11·7 (9·7,14·1)			
Shrubs/grass					28·1 (14·7,53·8)			

1. 'Other South Asia' includes Bangladesh and Pakistan (excludes India)
2. South America includes Chile and Colombia
3. Africa includes Tanzania and Zimbabwe

Table S11. Summary of average 48-hour BC absorbance levels ($10^{-5}m^{-1}$) among personal samples by primary fuel type.

	Primary Fuel Type															
	All Homes		Gas		Electric		Coal		Ag/crop waste		Wood		Animal dung		Shrub/ grass	
	Male	Female	Male	Female	Male	Female	Male	Female	Male	Female	Male	Female	Male	Female	Male	Female
Overall Mean (95% CI)	2·6 (2·3,2·9)	3·0 (2·7,3·3)	2·2 (1·9,2·6)	2·4 (2·1,2·7)	3·0 (2·5,3·7)	2·7 (2·1,3·4)	1·7 (1·3,2·3)	2·2 (1·7,3·0)	5·1 (3·3,8·0)	5·2 (3·7,7·3)	2·5 (2·0,3·0)	3·0 (2·6,3·5)	6·6 (3·8,11·3)	6·8 (4·4,10·7)	10·7 (6·7,17·1)	13·9 (9·1,21·4)
Country/ Region																
China	2·4 (2·2,2·7)	2·5 (2·2,2·8)	2·2 (1·9,2·7)	2·3 (1·9,2·9)	3·1 (2·6,3·8)	2·8 (2·2,3·5)	1·7 (1·2,2·3)	2·2 (1·6,2·9)	4·2 (2·6,6·7)	4·3 (3·0,6·1)	2·1 (1·7,2·6)	2·1 (1·6,2·7)	-	-	5·3 (1·1,25·9)	3·7 (1·5,9·4)
India	3·2 (2·6,3·9)	3·4 (2·9,4·0)	2·5 (2·0,3·3)	2·7 (2·2,3·3)	-	-			-		3·7 (2·4,5·6)	4·1 (3·0,5·5)	8·2 (7·6,8·7)	7·0 (5·1,9·6)	-	-
Other South Asia[1]	3·7 (2·3,5·8)	7·7 (5·4,11·0)	-	-	-	-	-	-	12·1 (8·7,16·9)	12·9 (9·4,17·7)	1·9 (1·2,2·8)	4·2 (2·6,6·9)	3·9 (0·5,32·9)	6·6 (1·5,29·5)	13·5 (11,16·5)	19·8 (15·7,25·1)
South America[2]	1·2 (0·8,1·6)	1·4 (1·0,1·8)	1·2 (0·8,1·9)	1·2 (0·9,1·6)	-	-	-	-	-	-	1·1 (0·7,1·7)	1·5 (1,2·2)	-	-	-	-
Africa[3]	3.0 (1.7,5.4)	3·5 (2·6,4·8)	-	-	-	1·4 (0·6,3·2)	-	-	-	-	3·0 (1·7,5·4)	3·8 (2·8,5·3)	-	-	-	-
Age																
43-60	3·3 (2·7,4·0)	4·2 (3·6,5·0)	2·7 (2·1,3·4)	2·6 (2·2,3·1)	2·8 (1·9,4·2)	2·6 (1·7,3·9)	1·6 (0·8,3·4)	2·1 (1·3,3·4)	6·1 (3·1,12·3)	8·1 (5·7,11·5)	3·3 (2·2,4·8)	3·6 (2·8,4·6)	10·0 (7·7,13·1)	8·7 (6,12·6)	3·3 (2·2,4·8)	20·2 (15·1,27·1)
61-84	2·4 (2·0,2·8)	2·9 (2·4,3·5)	1·9 (1·5,2·5)	2·1 (1·6,2·8)	3·4 (2·2,5·2)	2·8 (1·7,4·4)	1·5 (1·1,2·1)	2·2 (1·1,4·3)	4·5 (2·5,8·0)	4·5 (2·8,7)	2·3 (1·8,3)	2·5 (1·9,3·4)	8·0 (7·5,8·6)	4·0 (1·4,11·1)	2·3 (1·8,3)	7·4 (3·3,16·5)
Occupation air pollution exposure[4]																
Yes	2·8 (2·3,3·5)	2·9 (2·4,3·5)	2·5 (2·1,3·1)	2·0 (1·5,2·6)	3·5 (1·9,6·2)	1·4 (0·6,3·4)	2·8 (2·5,3·1)	-	-	2·5 (2·0,3·1)	3·0 (2·5,3·6)	8·3 (5,13·8)	4·8 (3·3,7)	2·5 (2,3·1)	18·7 (15·9,21·9)	2·5 (2·1,3·1)
No	2·5 (2·2,2·8)	3·0 (2·7,3·3)	2·1 (1·9,2·4)	2·5 (2·2,2·8)	3 (2·5,3·6)	2·8 (2·3,3·5)	1·6 (1·3,2·0)	2·2 (1·8,2·7)	4·9 (3·5,6·8)	5·2 (3·9,6·9)	2·4 (2·0,2·9)	3·0 (2·5,3·5)	6·0 (3·9,9·3)	8·0 (5·8,11·1)	2·4 (2·0,2·9)	12·9 (8·8,18·8)
Smoker																
Yes	2·9 (2·5,3·4)	3·1 (1·8,5·2)	2·8 (2·2,3·5)	4·3 (1·6,11·2)	3·2 (2·4,4·4)	-	2·0 (1·4,3·0)	-	4·3 (2·1,8·8)	-	2·4 (1·8,3·3)	2·7 (1·3,5·2)	8·4 (7·7,9·2)	-	2·4 (1·8,3·3)	-
No	2·4 (2·1,2·7)	3·0 (2·7,3·3)	1·9 (1·6,2·3)	2·4 (2,2·7)	2·9 (2·2,3·7)	2·7 (2·2,3·4)	1·6 (1·1,2·3)	2·2 (1·7,3·0)	5·7 (3·3,10·1)	5·5 (4·0,7·6)	2·5 (1·9,3·2)	3·0 (2·6,3·5)	6 (2·8,12·9)	6·8 (4·5,10·4)	2·5 (1·9,3·2)	14 (9·1,21·4)
2nd hand smoke exposure																
Yes	3·0 (2·5,3·4)	3·8 (3·3,4·5)	2·5 (2·1,3·0)	3·2 (2·6,4·1)	3·3 (2·4,4·4)	3·5 (2·6,4·7)	1·9 (1·3,2·7)	2·5 (1·8,3·4)	4·8 (3,7·9)	5·3 (3·3,8·6)	2·6 (2·2,3·2)	3·3 (2·6,4)	8·8 (7·2,10·8)	11·9 (7·0,20·0)	2·6 (2·2,3·2)	16·2 (12·7,20·7)
No	2·3 (2·0,2·6)	2·6 (2·3,2·9)	2·1 (1·8,2·4)	2·1 (1·8,2·4)	2·9 (2·4,3·5)	2·2 (1·7,2·9)	1·6 (1·3,2)	2·2 (1·7,2·8)	5·5 (3·9,7·9)	5·1 (3·6,7·3)	2·2 (1·8,2·7)	2·8 (2·4,3·4)	4·5 (1·5,13·0)	5·3 (3·8,7·4)	2·2 (1·8,2·7)	10·2 (4·3,24)

Among kitchen samples, BC levels were highly correlated with $PM_{2.5}$ kitchen concentrations (Spearman r~0·7-0·9) (Figure S15). The correlation between BC levels and $PM_{2.5}$ kitchen concentrations was generally highly in India (Spearman r~0·8-0·9) compared to China (Spearman r~0·65-0·8) (Figure S16). Within China, BC constituted a higher fraction of $PM_{2.5}$ in sub-national regions in western China (e.g. Inner Mongolia, Liaoning) than in eastern China (e.g. Shanxi, Sichuan). In India, $PM_{2.5}$ concentrations had higher BC content in northern India (Chandigarh) than all other sub-national Indian regions included in the study.

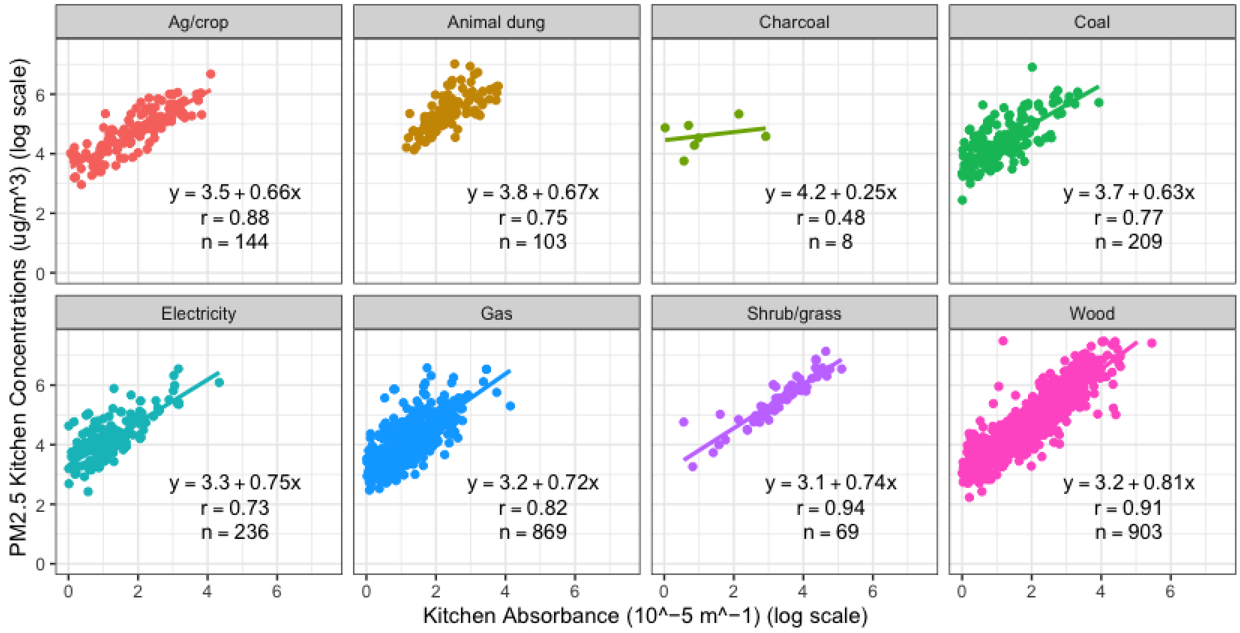

Figure S15. Relationship between BC kitchen absorbance (log scale) and $PM_{2.5}$ kitchen concentrations (log scale) in rural PURE-AIR communities by primary fuel type. Note: Regression equations are reported in units of µg/m^3 per 10^{-5}m^{-1}. r reported in the figure is the Spearman correlation.

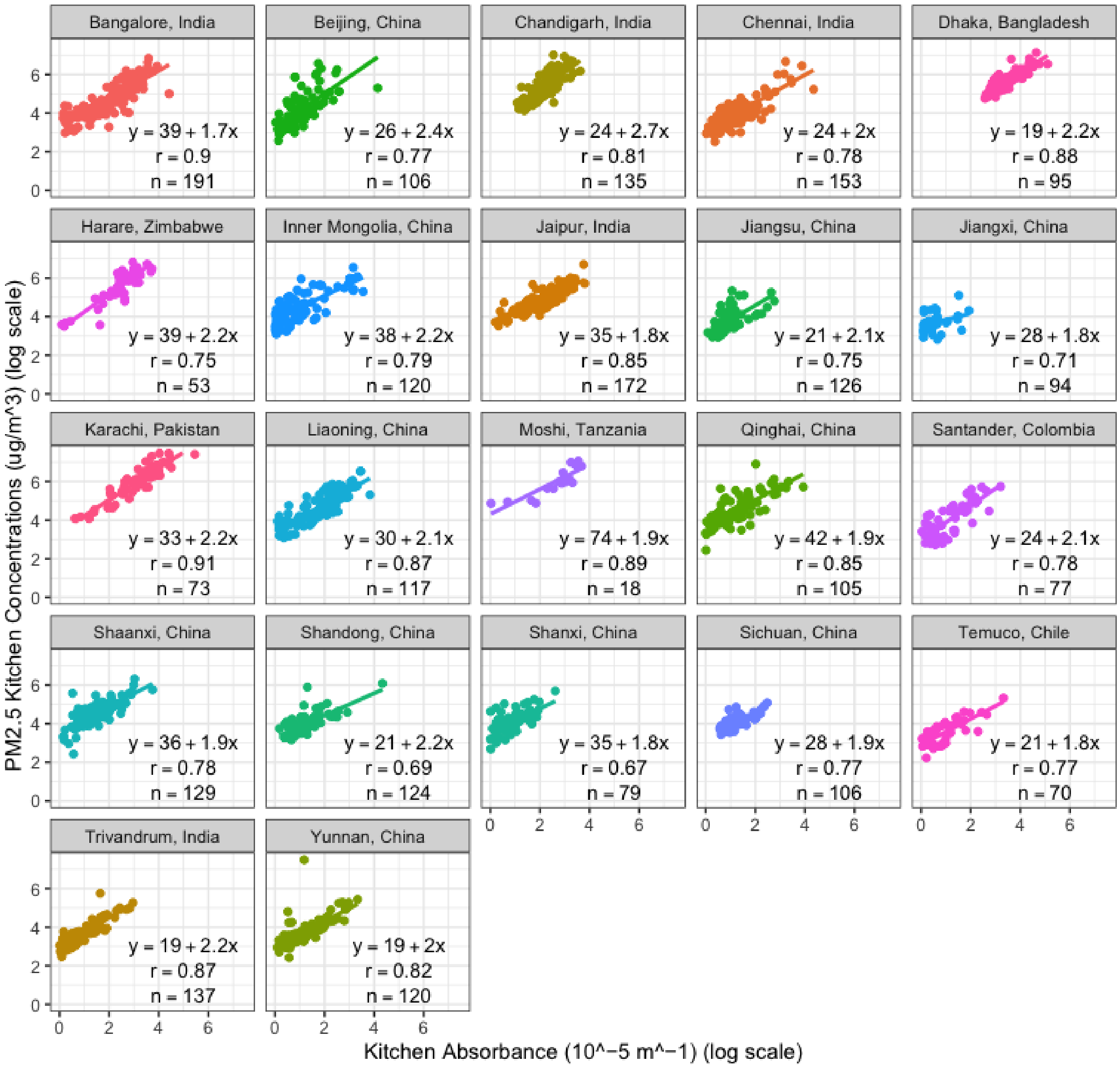

Figure S16. Relationship between BC kitchen absorbance (log scale) and $PM_{2.5}$ kitchen concentrations (log scale) in rural PURE-AIR communities by sub-national region. Note: Regression equations are reported in units of $\mu g/m^3$ per $10^{-5} m^{-1}$.

The relationship between black carbon (BC) and $PM_{2.5}$ concentrations was also modified by cooking location (indoor versus outdoor). In two sub-national regions of India (Jaipur, Chandigarh) where a mix of indoor and cooking occurred, outdoor kitchens had a much higher BC fraction of $PM_{2.5}$ (higher slope) compared to indoor kitchens (Figure S17). This supports the likelihood that the BC fraction of $PM_{2.5}$ was partially attributable to differences in ambient sources of BC, as the burning of crop residue in agricultural fields is common in India. In other countries with a combination of indoor/outdoor cooking areas (Colombia, Bangladesh, Tanzania), there were minor differences in the relationship between BC absorbance and $PM_{2.5}$ between indoor and outdoor cooking locations.

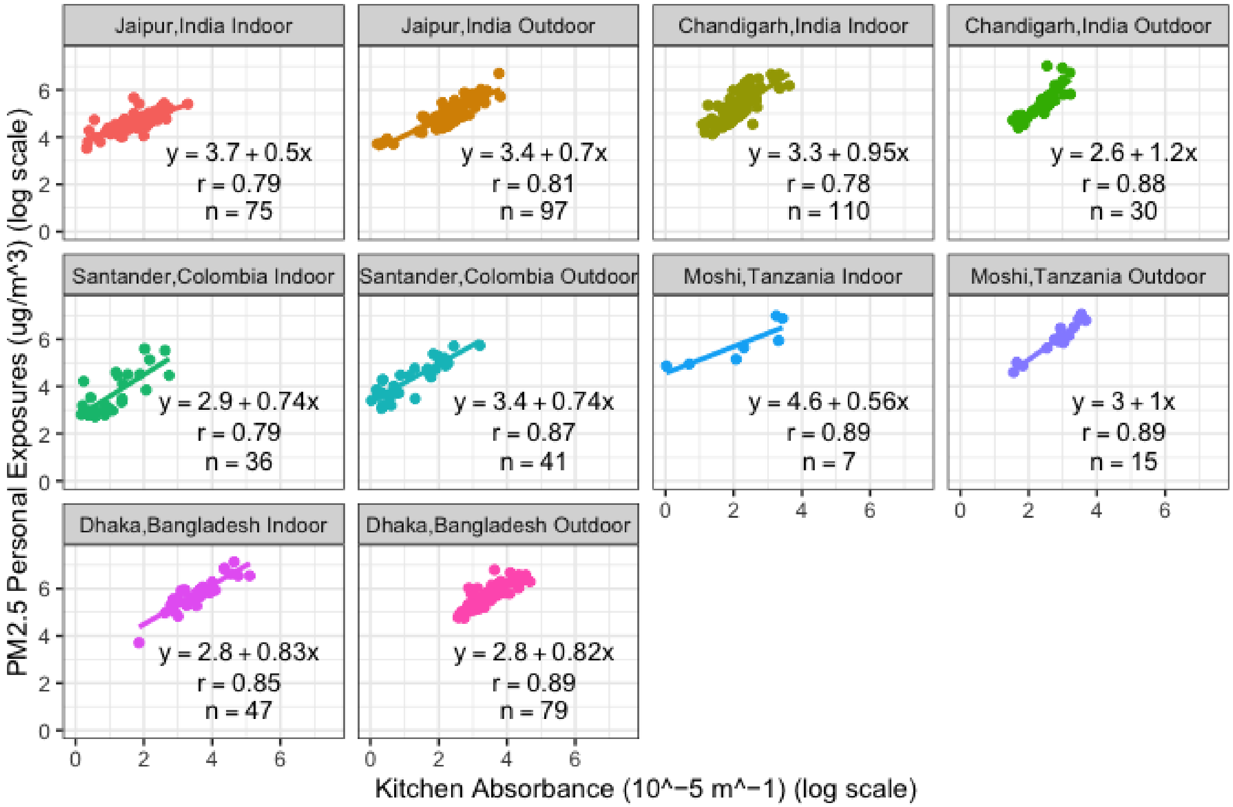

Figure S17. Relationship between BC absorbance and $PM_{2.5}$ in rural PURE-AIR communities by cooking location (indoor versus outdoor). Note: Regression equations are reported in units of $\mu g/m^3$ per $10^{-5} m^{-1}$.

BC kitchen absorbance was moderately correlated with BC personal absorbance (r=0·63) (Figure S18). The correlation was slightly lower than the correlation between $PM_{2.5}$ personal exposures and $PM_{2.5}$ kitchen concentrations (r=0·69) (Figure S12).

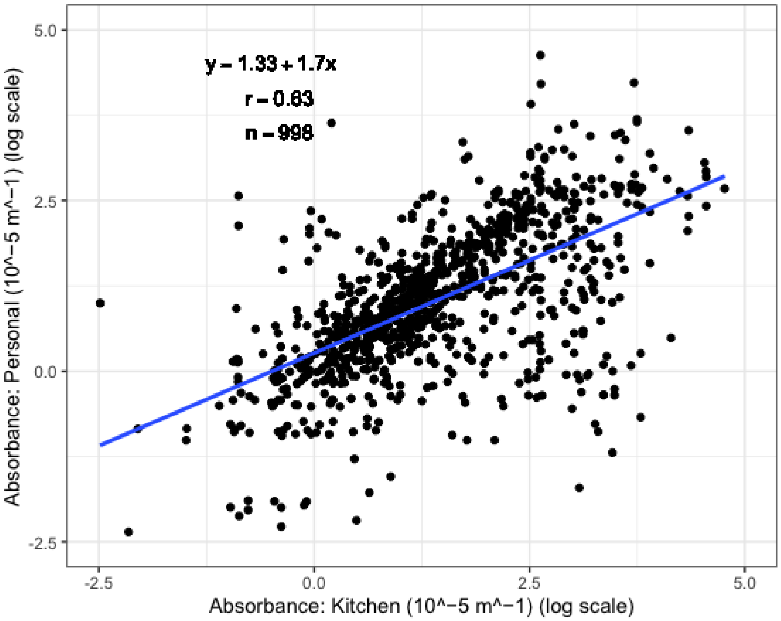

Figure S18. Relationship between kitchen absorbance and personal absorbance levels in rural PURE-AIR communities. Note: Regression equations are reported in units of $10^{-5}m^{-1}$.

Quantifying Between-Country Variability in HAP Levels

The intra-class correlation (ICC) was calculated to comparable the variability in average 48-hour $PM_{2.5}$ and BC kitchen concentrations and personal exposures at different levels of clustering in the PURE-AIR study (households nested in communities, nested in sub-national regions, nested within countries),

The ICC for $PM_{2.5}$ and BC kitchen measurements increased with increasing geographic level of aggregation (e.g. $ICC_{PM\text{-}Kitchen\text{-}community}$=0·27; $ICC_{PM\text{-}Kitchen\text{-}sub\text{-}national\text{-}region}$=0·55; $ICC_{PM\text{-}Kitchen\text{-}country}$=0·61 (Table S12). This indicates greater variability in average $PM_{2.5}$ and BC kitchen concentrations between PURE-AIR countries than within countries, in contrast with higher variability within communities than between communities.

Higher ICC at each geographic level among $PM_{2.5}$ personal measurements relative to the ICC of $PM_{2.5}$ kitchen concentrations potentially signals the influence of ambient $PM_{2.5}$ pollution levels on exposures. Ambient $PM_{2.5}$ pollution may be more dependent on national or regional factors than household concentrations from biomass burning due to varying levels of manufacturing/urbanization in the country and changes in meteorology that affect outdoor $PM_{2.5}$ levels. Conversely, lower ICC among personal BC absorbance measurements relative to kitchen measurements indicates localized BC emission sources (e.g. biomass burning, agricultural field burning) during PURE-AIR monitoring causing higher within-country and within-region variability in BC exposures.

Table S12. Intra-class correlations for 48-hour $PM_{2.5}$ and BC kitchen and personal measurements

Geographic Level	$ICC_{PM\text{-}Kitchen}$	$ICC_{BC\text{-}Kitchen}$	$ICC_{PM\text{-}Personal}$	$ICC_{BC\text{-}Personal}$
Community	0·27	0·27	0·39	0·27
Sub-national region	0·55	0·52	0·61	0·38
Country	0·61	0·59	0·68	0·38

Socioeconomic Status

On a multinational level, increasing education level and household asset index were associated with significantly lower (non-overlapping confidence intervals) average $PM_{2.5}$ kitchen concentrations (independent of primary cooking fuel type) (Figure S19). The average 48-hour $PM_{2.5}$ kitchen concentration in households with members having secondary school education (61 μg/m^3 95%CI:[57,65]) was 10 and 44 μg/m^3 lower than that among households with members having primary education (71 μg/m^3 95%CI:[67,76]) and no formal education (107 μg/m^3 95%CI:[99,115]), respectively. The average 48-hour $PM_{2.5}$ kitchen concentration in households in the highest household asset index tertile (62 μg/m^3 95%CI:[55,70]) was 15 and 17 μg/m^3 lower than that among households in the middle (77 μg/m^3 95%CI:[71,82]) and lowest tertile (79 μg/m^3 95%CI:[75,83]), respectively.

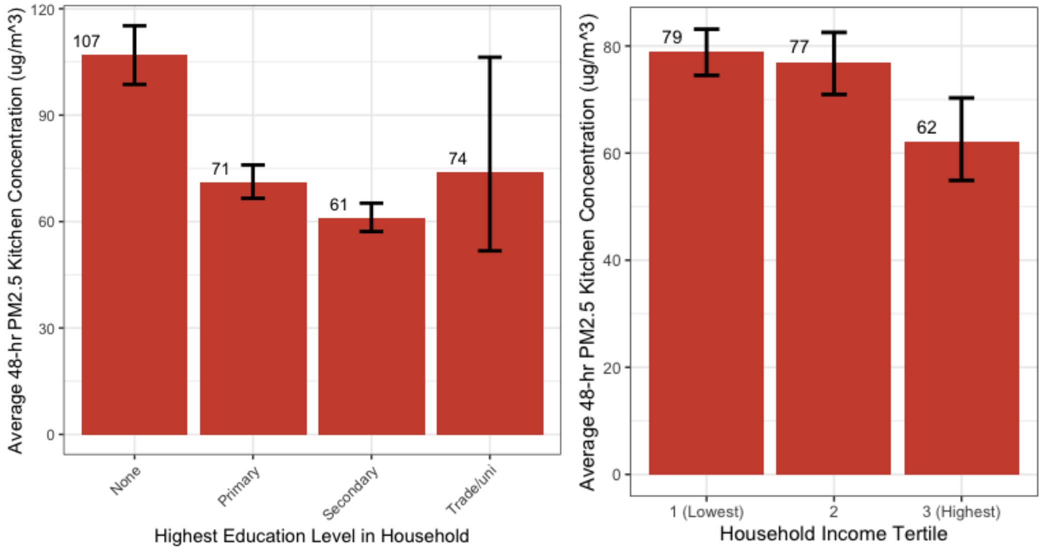

Figure S19. Association of highest household education level (left) and household asset index (right) with average 48-hour $PM_{2.5}$ kitchen concentrations in rural PURE-AIR communities by country

The strength of association between education/household income with kitchen concentrations varied between and within-countries (Figure S20); higher education level had a low, negative association with $PM_{2.5}$ concentrations in China and a high, negative association in India, South American and African countries. The same negative relationship between increasing household asset index tertile and $PM_{2.5}$ kitchen concentrations held in most countries (except China, Bangladesh, Pakistan) (Figure S21).

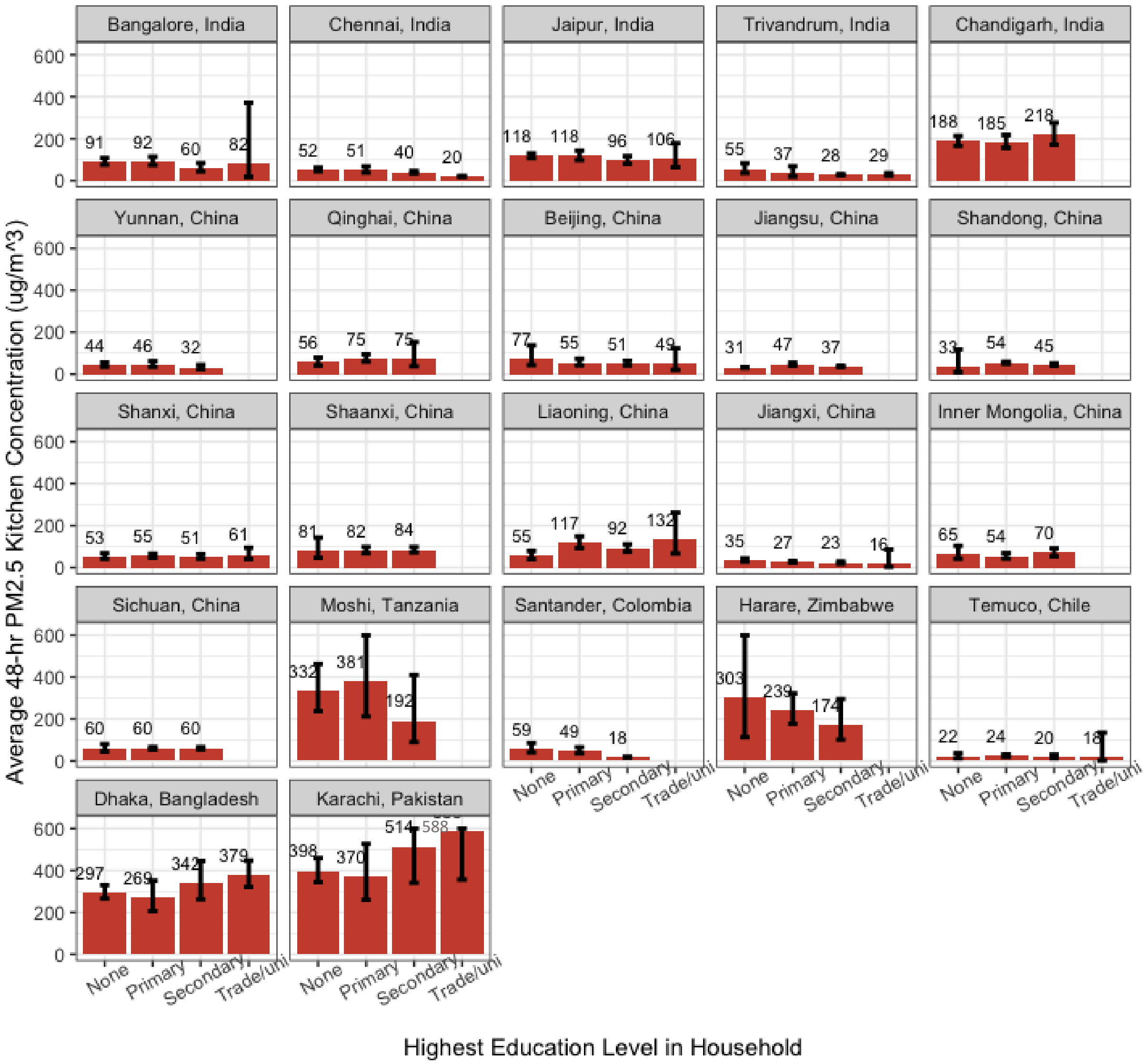

Figure S20. Association of highest household education level with average 48-hour $PM_{2.5}$ kitchen concentrations in rural PURE-AIR communities by sub-national region

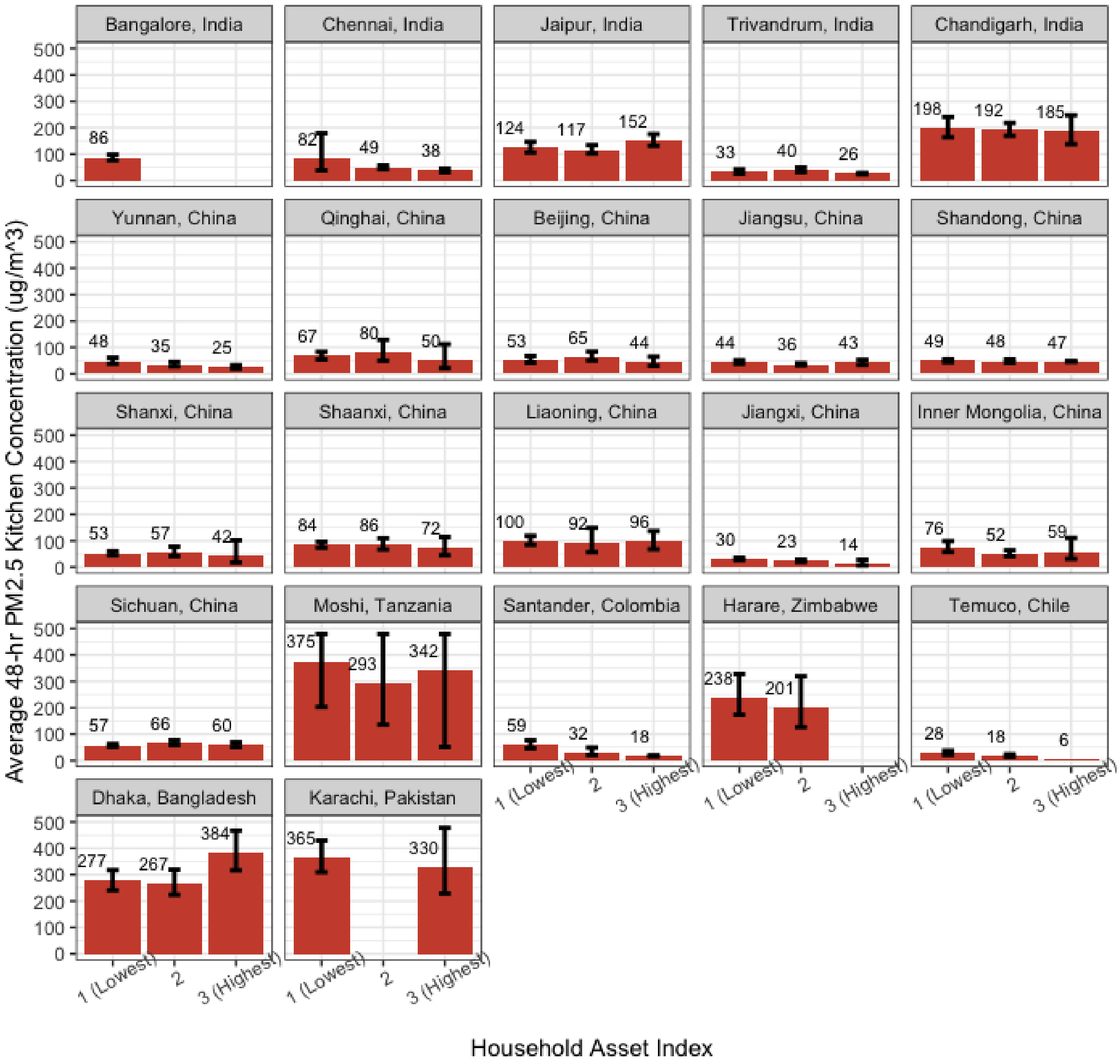

Figure S21. Association of household asset index with average 48-hour $PM_{2.5}$ kitchen concentrations in rural PURE-AIR communities by sub-national region

A previous analysis examining predictors of primary fuel switching among the same study sample similarly revealed that household-level SES factors were also stronger determinants of polluting-to-clean fuel switching in India compared to China.[1] Improving access to education for women, particularly in sub-Saharan Africa, may offer robust reductions in HAP exposures; higher education level was 5-10 times more strongly associated with HAP-$PM_{2.5}$ concentration reductions than increasing household asset index in African PURE rural communities.

A positive association between increasing household asset index and HAP-$PM_{2.5}$ concentrations in many sub-national regions in China signals that the relationship between household SES may and HAP exposures in more developed countries may be confounded by sources of ambient air pollution.

GPS Data

GPS data obtained from the UPAS was use to dichotomize all personal exposure measurements as ‘at home’ or ‘away from home’ based on whether the participant was <=25 or >25 meter radius away from the household, respectively. Using this method, a sub-national region and sex-specific mean ‘percent of time away from home’ were calculated during the 48-hour monitoring. Overall mean time spent away from home during the 48-hour

monitoring was 11.9 hours (range among sub-national regions: 6.6-18.8 hours) (Table S13). Only 5% (n=45) of participants did not spend time away from their household during 48-hour sampling (Table S13), which potentially suggests high compliance with personal monitoring.

Table S13. Hours spent away from home during the 48-hour monitoring by PURE-AIR sub-national region and sex

Sub-National Region	Sex	% Time Away From Home (Mean)	% Time Away From Home (SD)	Hours Away From Home (Mean)	% of participants with 0% Time Away From Home
Beijing, China	Female	23.1	16.7	11.1	0
Beijing, China	Male	18.0	15.5	8.6	7
Chennai, India	Female	19.6	18.2	9.4	9
Chennai, India	Male	17.0	15.2	8.1	9
Dhaka, Bangladesh	Female	29.5	22.9	14.2	8
Dhaka, Bangladesh	Male	18.2	18.1	8.8	10
Harare, Zimbabwe	Female	15.5	11.2	7.4	10
Harare, Zimbabwe	Male	25.3	16.0	12.1	6
Inner Mongolia, China	Female	26.9	23.7	12.9	6
Inner Mongolia, China	Male	21.3	19.1	10.2	0
Jiangsu, China	Female	27.4	19.3	13.1	0
Jiangsu, China	Male	30.3	18.9	14.6	0
Jiangxi, China	Female	36.2	21.4	17.4	13
Jiangxi, China	Male	27.2	19.4	13.0	0
Karachi, Pakistan	Female	13.8	12.1	6.6	0
Karachi, Pakistan	Male	22.1	19.4	10.6	8
Liaoning, China	Female	28.0	19.9	13.4	0
Liaoning, China	Male	33.5	20.8	16.1	0
Moshi, Tanzania	Female	35.4	20.6	17.0	0
Moshi, Tanzania	Male	25.2	11.5	12.1	0
Qinghai, China	Female	21.9	19.3	10.5	0
Qinghai, China	Male	20.3	14.2	9.7	0
Santander, Colombia	Female	15.2	13.0	7.3	7
Santander, Colombia	Male	18.0	11.7	8.7	12
Shaanxi, China	Female	21.7	19.2	10.4	0
Shaanxi, China	Male	20.9	16.5	10.0	0
Shandong, China	Female	26.4	18.8	12.7	5
Shandong, China	Male	15.5	14.9	7.5	11
Shanxi, China	Female	25.3	17.4	12.2	0
Shanxi, China	Male	26.2	16.9	12.6	10
Sichuan, China	Female	36.7	18.6	17.6	0
Sichuan, China	Male	27.6	15.0	13.2	0
Temuco, Chile	Female	32.2	8.2	15.4	0
Temuco, Chile	Male	39.2	17.8	18.8	0
Trivandrum, India	Female	37.9	17.3	18.2	0
Trivandrum, India	Male	29.3	20.1	14.1	0
Yunnan, China	Female	16.0	14.6	7.7	11
Yunnan, China	Male	21.7	18.3	10.4	14

Note: GPS data not available for all personal samples as participants had the option to opt out of GPS monitoring.

Funding/Support:

Dr S Yusuf is supported by the Mary W Burke endowed chair of the Heart and Stroke Foundation of Ontario.

The PURE study is an investigator-initiated study that is funded by the Population Health Research Institute, Hamilton Health Sciences Research Institute (HHSRI), the Canadian Institutes of Health Research, Heart and Stroke Foundation of Ontario, Support from Canadian Institutes of Health Research's Strategy for Patient Oriented Research, through the Ontario SPOR Support Unit, as well as the Ontario Ministry of Health and Long-Term Care and through unrestricted grants from several pharmaceutical companies [with major contributions from AstraZeneca (Canada), Sanofi-Aventis (France and Canada), Boehringer Ingelheim (Germany and Canada), Servier, and GlaxoSmithKline], and additional contributions from Novartis and King Pharma and from various national or local organisations in participating countries.

These include: **Argentina:** Fundacion ECLA **(Estudios Clínicos Latino America)** ; **Bangladesh**: Independent University, Bangladesh and Mitra and Associates; **Brazil:** Unilever Health Institute, Brazil; **Canada:** This study was supported by an unrestricted grant from Dairy Farmers of Canada and the National Dairy Council (U.S.), Public Health Agency of Canada and Champlain Cardiovascular Disease Prevention Network; **Chile:** Universidad de La Frontera [DI13-PE11]; **China:** National Center for Cardiovascular Diseases and ThinkTank Research Center for Health Development; **Colombia:** Colciencias (grant 6566-04-18062 and grant 6517-777-58228); **India:** Indian Council of Medical Research; **Malaysia:** Ministry of Science, Technology and Innovation of Malaysia (grant number: 100-IRDC/BIOTEK 16/6/21 [13/2007], and 07-05-IFN-BPH 010), Ministry of Higher Education of Malaysia (grant number: 600-RMI/LRGS/5/3 [2/2011]), Universiti Teknologi MARA, Universiti Kebangsaan Malaysia (UKM-Hejim-Komuniti-15-2010); **occupied Palestinian territory:** the United Nations Relief and Works Agency for Palestine Refugees in the Near East, occupied Palestinian territory; International Development Research Centre, Canada; **Philippines:** Philippine Council for Health Research and Development; **Poland:** Polish Ministry of Science and Higher Education (grant number: 290/W-PURE/2008/0), Wroclaw Medical University; **Saudi Arabia:** Saudi Heart Association. Saudi Gastroenterology Association. Dr.Mohammad Alfagih Hospital. The Deanship of Scientific Research at King Saud University, Riyadh, Saudi Arabia (Research group

number: RG -1436-013); **South Africa:** The North-West University, SA and Netherlands Programme for Alternative Development, National Research Foundation, Medical Research Council of South Africa, The South Africa Sugar Association, Faculty of Community and Health Sciences; **Sweden:** Grants from the Swedish state under the Agreement concerning research and education of doctors; the Swedish Heart and Lung Foundation; the Swedish Research Council; the Swedish Council for Health, Working Life and Welfare, King Gustaf V:s and Queen Victoria Freemason's Foundation, AFA Insurance; **Turkey:** Metabolic Syndrome Society, AstraZeneca, Sanofi Aventis; **United Arab Emirates:** Sheikh Hamdan Bin **Role of Sponsor:** The external Rashid Al Maktoum Award For Medical Sciences and Dubai Health Authority, Dubai.

funders and sponsors had no role in the design and conduct of the study; in the collection, analysis, and interpretation of the data; in the preparation, review, or approval of the manuscript; or in the decision to submit the manuscript for publication.

PURE Project Office Staff, National Coordinators, Investigators, and Key Staff:

Project office (Population Health Research Institute, Hamilton Health Sciences and McMaster University, Hamilton, Canada): S Yusuf* (Principal Investigator).
S Rangarajan (Program Manager); K K Teo, S S Anand, C K Chow, M O'Donnell, A Mente, D Leong, A Smyth, P Joseph, M Duong, O Kurmi, R D'Souza, M Walli-Attaei, B Balaji, R Naito, S Islam (Statistician), W Hu (Statistician), C Ramasundarahettige (Statistician), P Sheridan (Statistician), S Bangdiwala, L Dyal, M Dehghan (Nutrition Epidemiologist), A Aliberti, A Reyes, A Zaki, B Connolly, B Zhang, D Agapay, D Krol, E McNeice, E Ramezani, F Shifaly, G McAlpine, I Kay, J Rimac, J Swallow, M Di Marino, M Jakymyshyn, M(a) Mushtaha, M(o) Mushtaha, M Trottier, N Aoucheva, N Kandy, P Mackie, R Buthool, R Patel, R Solano, S Gopal, S Ramacham, S Trottier

Core Laboratories: G Pare, M McQueen, S Lamers, J Keys (Hamilton), X Wang (Beijing, China), A Devanath (Bangalore, India).
Argentina: R Diaz*, A Orlandini, P Lamelas, M L Diaz, A Pascual, M Salvador, C Chacon;
Bangladesh: O Rahman*, R Yusuf*, S A K S. Ahmed, T Choudhury, M Sintaha, A Khan, O

Alam, N, Nayeem, S N Mitra, S Islam, F Pasha; **Brazil:** A Avezum*, C S Marcilio, A C Mattos, G B Oliveira; **Canada:** K Teo***,** S Yusuf*****, Sumathy Rangarajan, A Arshad, B Bideri, I Kay, J Rimac, R Buthool, S Trottier, G Dagenais, P Poirier, G Turbide, AS Bourlaud, A LeBlanc De Bluts, M Cayer, I Tardif, M Pettigrew, S Lear, V de Jong, A N Saidy, V Kandola, E Corber, I Vukmirovich, D Gasevic, A Wielgosz, A Pipe, A Lefebvre, A Pepe, A Auclair, A Prémont, A S Bourlaud; **Chile:** F Lanas*, P Serón, M J Oliveros, F Cazor, Y Palacios; **China:** Liu Lisheng*, Li Wei*, Chen Chunming#, Zhao Wenhua. Hu Bo, Yin Lu, Zhu Jun, Liang Yan, Sun Yi, Wang Yang, Deng Qing, Jia Xuan, He Xinye, Zhang Hongye, Bo Jian, Wang Xingyu, Liu Xu, Gao Nan, Bai Xiulin, Yao Chenrui, Cheng Xiaoru, Wang Chuangshi, Li Sidong, Liu Weida, Lang Xinyue, Liu Xiaoyun, Zhu Yibing, Xie Liya, Liu Zhiguang, Ren Yingjuan, Dai Xi, Gao Liuning, Wang Liping, Su yuxuan, Han Guoliang, Song Rui, Cao Zhuangni, Sun Yaya, Li Xiangrong, Wang Jing, Wang Li, Peng Ya, Li Xiaoqing, Li Ling, Wang Jia, Zou Jianmei, Gao Fan, Tian Shaofang, Liu Lifu, Li Yongmei, Bi Yanhui, Li Xin, Zhang Anran, Wu Dandan, Cheng ying, Xiao Yize, Lu Fanghong, Li Yindong, Hou Yan, Zhang Liangqing, Guo Baoxia, Liao Xiaoyang, Chen Di, Zhang Peng, Li Ning, Ma Xiaolan, Lei Rensheng, Fu Minfan, Liu Yu, Xing Xiaojie, Yang Youzhu, Zhao Shenghu, Xiang Quanyong, Tang Jinhua, Liu Zhengrong, Qiang Deren, Li Xiaoxia, Xu Zhengting, Aideeraili.Ayoupu, Zhao Qian; **Colombia:** P Lopez-Jaramillo*, P A Camacho-Lopez, M Perez, J Otero-Wandurraga, D I Molina, C Cure-Cure, JL Accini, E Hernandez, E Arcos, C Narvaez, A Sotomayor, F Manzur, H Garcia, G Sanchez, F Cotes, A Rico, M Duran, C Torres; **India: Bangalore -** P Mony *, M Vaz*, S Swaminathan, AV Bharathi, K Shankar, A V Kurpad, K G Jayachitra, H A L Hospital, AR Raju, S Niramala, V Hemalatha, K Murali, C Balaji, A Janaki, K Amaranadh, P Vijayalakshmi, **Chennai** - V Mohan*, R M Anjana, M Deepa, K Parthiban, L Dhanasekaran, SK Sundaram, M Rajalakshmi, P Rajaneesh, K Munusamy, M Anitha, S Hemavathy, T Rahulashankiruthiyayan, D Anitha, R. Dhanasekar, S. Sureshkumar, D Anitha, K Sridevi, **Jaipur** - R Gupta, R B Panwar, I Mohan, P Rastogi, S Rastogi, R Bhargava, M Sharma, D Sharma, **Trivandrum -** V Raman Kutty, K Vijayakumar, S Nair, Kamala R, Manu MS, Arunlal AR, Veena A, Sandeep P Kumar, Leena Kumari, Tessi R, Jith S, K Ajayan, G Rajasree, AR Renjini, A Deepu, B Sandhya, S Asha, H S Soumya, **Chandigarh**- R Kumar, M Kaur, P V M Lakshmi, V Sagar J S Thakur, B Patro, R Mahajan, A Josh, G Singh, K Sharma, P Chaudary, **Iran:** R Kelishadi*, A Bahonar, N Mohammadifard, H Heidari, **Kazakhstan:** K Davletov*, B Assembekov, B Amirov; **Kyrgyzstan:** E Mirrakhimov*,

S Abilova, U Zakirov, U Toktomamatov; **Malaysia: UiTM -** K Yusoff*, T S Ismail, K Ng, A Devi, N Mat-Nasir, AS Ramli, MNK Nor-Ashikin, R Dasiman, MY Mazaouspavina, F Ariffin, M Miskan, H Abul-Hamid, S Abdul-Razak, N Baharudin, NMN Mohd-Nasir, SF Badlishah-Sham, M Kaur, M Koshy, F A Majid, N A Bakar, N Zainon, R Salleh, SR Norlizan, NM Ghazali, M Baharom, H Zulkifli, R Razali, S Ali, CWJCW Hafar, F Basir; **UKM** - Noorhassim Ismail, M J Hasni, M T Azmi, M I Zaleha, R Ismail, K Y Hazdi, N Saian, A Jusoh, N Nasir, A Ayub, N Mohamed, A Jamaludin, Z Rahim; **Occupied Palestinian Territory:** R Khatib*, U Khammash, R Giacaman; **Pakistan:** R Iqbal*, R Khawaja, I Azam, K Kazmi; **Peru:** J Miranda*, A Bernabe Ortiz, W Checkley, R H Gilman, L Smeeth, R M Carrillo, M de los Angeles, C Tarazona Meza**; Philippines:** A Dans*, H U Co, J T Sanchez, L Pudol, C Zamora-Pudol, L A M Palileo-Villanueva, M R Aquino, C Abaquin, SL Pudol, K Manguiat, S Malayang; **Poland:** W Zatonski*, A Szuba, K Zatonska, R Ilow#, M Ferus, B Regulska-Ilow, D Różańska, M Wolyniec; **Saudi Arabia:** KF AlHabib*, M Alshamiri, HB Altaradi, O Alnobani, N Alkamel, M Ali, M Abdulrahman, R Nouri; **South Africa:** L Kruger*, A Kruger#, P Bestra, H Voster, A E Schutte, E Wentzel-Viljoen, FC Eloff, H de Ridder, H Moss, J Potgieter, A Roux, M Watson, G de Wet, A Olckers, J C Jerling, M Pieters, T Hoekstra, T Puoane, R Swart*, E Igumbor, L Tsolekile, K Ndayi, D Sanders, P Naidoo, N Steyn, N Peer, B Mayosi#, B Rayner, V Lambert, N Levitt, T Kolbe-Alexander, L Ntyintyane, G Hughes, J Fourie, M Muzigaba, S Xapa, N Gobile , K Ndayi, B Jwili, K Ndibaza, B Egbujie; **Sweden** A Rosengren*, K Bengtsson Boström, A Rawshani, A Gustavsson, M Andreasson, L Wirdemann; **Tanzania:** K Yeates*, M Oresto, N West **Turkey:** A Oguz*, N Imeryuz, Y Altuntas, S Gulec, A Temizhan, K Karsidag, K B T Calik, A K Akalin, O T Caklili, M V Keskinler, K Yildiz; **United Arab Emirates:** A H Yusufali, F Hussain, M H S Abdelmotagali, D F Youssef, O Z S Ahmad, F H M Hashem, T M Mamdouh, F M AbdRabbou, S H Ahmed, M A AlOmairi, H M Swidan, M Omran, N A Monsef ; **Zimbabwe:** J Chifamba*, T Ncube, B Ncube, C Chimhete, G K Neya, T Manenji, L Gwaunza, V Mapara, G Terera, C Mahachi, P Murambiwa, R Mapanga, A Chinhara

*National Coordinator

Deceased

PURE Country Institution Names:

	**Institution**
**South Africa**	Faculty of Health Science<br>North-West University<br>Potchefstroom Campus
	University of the Western Cape<br>Department of Dietetics and Nutrition<br>Private Bag X17, 7535<br>Bellville, South Africa
**Zimbabwe**	University of Zimbabwe<br>College of Health Sciences<br>Physiology Department<br>Harare, Zimbabwe
**Tanzania**	Pamoja Tunaweza Women Center, Moshi, Tanzania<br>Division of Nephrology, Department of Medicine<br>Queen's University
**China**	National Centre for Cardiovascular Diseases<br>Cardiovascular Institute & Fuwai Hospital<br>Chinese Academy of Medical Sciences<br>167, Bei Li Shi Lu, Beijing, China
	Fuwai Hospital<br>167 Beilishi Rd. Xicheng District<br>Beijing. 100037 China
**Philippines**	University of Philippines, Section of Adult Medicine & Medical Research Unit, Manila, Philippines
**Pakistan**	Department of Community Health Sciences and Medicine<br>Aga Khan University<br>Stadium Road, P.O Box 3500<br>Karachi Pakistan
**India, Bangalore**	St John's Medical College and Research Institute<br>Bangalore 560034, India
**India,**	Madras Diabetes Research

**Chennai**	Foundation &<br>Dr. Mohan's Diabetes Specialities Centre, Chennai
**India Jaipur**	Eternal Heart Care Centre and Research Institute, Jaipur
**India, Trivandrum**	Health Action by People, Thiruvananthapuram, Kerala, 695011 INDIA
**India, Chandigarh**	School of Public Health, Post Graduate Institute of Medical Education & Research, Chandigarh (India)
**Bangladesh**	Independent University, Bangladesh<br>Bashundhara, Dhaka<br>Bangladesh
**Malaysia**	Universiti Teknologi MARA, Sungai Buloh, Selangor, Malaysia AND UCSI University, Cheras, Selangor, Malaysia
	Department of Community Health. Faculty of Medicine. University Kebangsaan Malaysia. Kuala Lumpur. Malaysia
**Poland**	Wroclaw Medical University Department of Internal Medicine; Department of Social Medicine<br>Borowska 213 street; 50- 556 Wroclaw, Poland
	Department of Epidemiology,<br>The Maria Skłodowska-Curie Memorial Cancer Center and Institute of Oncology<br>02-034 Warsaw, 15B Wawelska str.<br>Poland
**Turkey**	Istanbul Medeniyet University<br>Istanbul, Turkey
**Sweden**	Sahlgrenska Academy<br>University of Gothenburg<br>Sweden

**Iran**	Isfahan Cardiovascular Research Center, Isfahan Research Institute<br>Isfahan University of Medical Sciences, Isfahan, Iran
**UAE**	Dubai Medical University, Hatta Hospital, Dubai Health Authority, Dubai, United Arab Emirates
**Saudi Arabia**	Department of Cardiac Sciences, King Fahad Cardiac Center<br>College of Medicine<br>King Saud University<br>Riyadh, Saudi Arabia
**Palestine**	Institute of Community and Public Health, Birzeit University, Ramallah, occupied Palestinian territory
**Canada**	Université Laval Institut universitaire de cardiologie et de pneumologie de Québec, Quebec<br>Canada G1V 4G5
	Simon Fraser University, Dept. of Biomedical Physiology & Kinesiology, BC, Canada
	Department of Medicine, University of Ottawa, Ottawa, Canada
	Population Health Research Institute, McMaster University, Hamilton Health Sciences, Hamilton, Ontario, Canada
**Argentina**	Estudios Clinicos Latinoamerica ECLA<br>Rosario, Santa Fe<br>Argentina<br><br>Department of Chronic Diseases<br>South American Center of Excellence for

	Cardiovascular Health (CESCAS)<br>Institute for Clinical Effectiveness and Health Policy (IECS)
**Brazil**	Dante Pazzanese Institute of Cardiology;<br>Hospital Alemao Oswaldo Cruz<br>Sao Paulo, SP Brazil
**Colombia**	Facultad de Ciencias de la Salud, Universidad de Santander (UDES), Bucaramanga, Santander, Fundacion Oftalmologica de Santander (FOSCAL) Floridablanca-Santander, Colombia
**Chile**	Universidad de La Frontera Temuco, Chile
**Ecuador**	DECANO<br>Facultad de Ciencias de la Salud Eugenio Espejo<br>Universidad Tecnológica Equinoccial<br>Dirección: Av. Mariscal Sucre s/n y Av. Mariana de Jesús, Quito Ecuador
**Peru**	CRONICAS Centro de Excelencia en Enfermedades Crónicas | www.cronicas-upch.pe<br>Universidad Peruana Cayetano Heredia | www.upch.edu.pe<br>Av. Armendáriz 497, Miraflores, Lima
**Russia**	Research Institute for Complex Issues of Cardiovascular Diseases, Kemerovo, Russia<br><br>Institute For Medical Education, Yaroslav-the-Wise Novgorod State University Ministry of

	Education and Science of the Russian Federation<br>Russia, Saint-Petersburg, 197022,<br>Karpovka river emb., Bld.13, office 28
**Kazakhstan**	Research Institute of Cardiology & Internal Diseases, Almaty, Kazakhstan
**Kyrgyzstan**	Kyrgyz Society of Cardiology, National Center of Cardiology and Internal Disease, Bishkek, Kyrgyzstan

References

1. Shupler M, Hystad P, Gustafson P, Rangarajan S, Mushtaha M, Jayachtria KG, et al. Household, community, sub-national and country-level predictors of primary cooking fuel switching in nine countries from the PURE study. Environmental Research Letters. 2019 Jul 29;14(8):085006.

2. Shupler M, Godwin W, Frostad J, Gustafson P, Arku RE, Brauer M. Global estimation of exposure to fine particulate matter (PM2.5) from household air pollution. Environment International. 2018 Nov;120:354–63.
